# A male-pheromone-elevated transcription factor ZNF362.1 in female schistosomes determines sexual maturation

**DOI:** 10.1126/sciadv.aec6907

**Published:** 2026-03-06

**Authors:** Mengjie Gu, Wenjun Cheng, Shan Li, Gongwen Chen, Xu Chen, Ruiqi Jiang, Minwei Yuan, Jing Wang, Wei Zhang, Cun Yi, Yuxiang Xie, Xiaoling Wang, Wei Hu, Jipeng Wang

**Affiliations:** ^1^ State Key Laboratory of Genetics and Development of Complex Phenotypes, Ministry of Education Key Laboratory of Contemporary Anthropology, School of Life Sciences, Fudan University, Shanghai, China.; ^2^ College of Life Sciences, Inner Mongolia University, Hohhot, Inner Mongolia Autonomous Region, China.

## Abstract

Egg production by female schistosomes drives both transmission and pathology of schistosomiasis, affecting over 200 million people. Female maturation relies on the male-derived pheromone β-alanyl-tryptamine (BATT), but underlying molecular mechanisms are unclear. We identified the BATT-responsive transcription factor gene *znf362* as a key regulator of female reproductive development. Functional studies showed that *znf362.1*, but not *znf362.2*, is essential for BATT-induced ovary and vitellaria maturation. Single-cell transcriptomics and in situ hybridization revealed up-regulation of *znf362.1* in oocytes and vitellaria S_1_ cells after BATT exposure. Multiomics analysis showed ZNF362.1 directly activates *Smp_349410*, a female gonad-specific gene encoding a CPEB1 homolog. Loss of *znf362.1* or *Smp_349410* impaired oocyte and vitellocyte differentiation without affecting progenitors. Mechanistically, *Sm*CPEB1 promotes female ovary development by regulating polyadenylation of cyclin B1 mRNA and drives S_1_ cell differentiation in the vitellaria. These findings define a transcriptional and post-transcriptional axis, BATT-*znf362.1*-*cpeb1*, that initiates female sexual maturation, offering mechanistic insight into schistosome reproduction and potential targets for schistosomiasis control.

## Teaser

Female *Schistosoma mansoni* start sexual maturation upon male signals, highlighting potential therapy targets.

## INTRODUCTION

Schistosomiasis, caused by parasitic flatworms of the genus *Schistosoma*, is a major zoonotic disease affecting over 200 million people across 78 countries (*1*). The primary agents of pathogenesis and transmission are the eggs produced by sexually mature female worms. A single adult female can produce hundreds to over a thousand eggs per day, depending on the species (*2*), which become lodged in host tissues such as the liver, mesentery and urogenital organs. As these eggs develop, they release antigens that elicit a host immune response, initiating granulomatous inflammation and, over time, causing severe chronic pathologies including liver fibrosis, portal hypertension, splenomegaly, and ascites (*3*, *4*). In contact with freshwater, eggs excreted via feces or urine hatch into miracidia, which infect intermediate snail hosts and perpetuate the parasite’s life cycle.

The sexual maturation of female schistosomes is a prerequisite for egg production. Two specialized reproductive organs are involved: the ovary, which generates mature oocytes, and the vitellaria, which produce eggshell precursors and nutritional material for the developing embryo (*5*). As dioecious flatworms, a rarity among Platyhelminths, schistosomes exhibit a unique mode of reproduction. Notably, female schistosomes cannot complete the development of their reproductive organs in the absence of a male. Instead, continuous physical pairing with the male worm, via his gynecophoral canal, is essential to trigger and maintain female sexual maturation. This phenomenon of male-induced female development was first described in 1928 (*6*). More recently, a dipeptide pheromone synthesized by male worms, β-alanyl-tryptamine (BATT), was identified as the key molecular cue responsible for initiating this developmental process in females (*7*). However, the downstream mechanisms by which female schistosomes interpret and respond to BATT signals to initiate sexual maturation remain largely unknown.

Adult *Schistosoma* parasites comprise more than 70 distinct cell types across various tissues (*8*). Organ development is governed by the differentiation of stem cells, not only within somatic tissues but also in the germline. In virgin females that have never paired with a male, the ovary and vitellaria are populated predominantly by undifferentiated stem-like cells, germline stem cells (GSCs) in the ovary and S_1_ cells in the vitellaria (*8*, *9*). Upon pairing with males, these progenitor cells undergo differentiation into mature cell types that constitute fully functional reproductive organs. This developmental process is reversible: When adult females are separated from their male partners, their ovaries and vitellaria gradually regress to an immature, undifferentiated state (*10*). Transcription factors (TFs) are pivotal regulators in organogenesis by orchestrating gene expression programs that regulate cell fate determination, differentiation, morphogenesis, and tissue patterning (*11*–*13*). Recent studies have identified several TFs involved in schistosome sexual development, including *onecut-1*, which governs GSC fate in male testes (*14*); *vitellogenetic factor 1*, required for vitellaria maturation (*15*); and *retinoic acid receptor* (*rar*), which regulates oocyte differentiation (*16*). However, these factors appear to function in a tissue-specific manner and do not explain how both female reproductive organs are simultaneously induced by BATT. This suggests the existence of a common upstream regulator that coordinates ovary and vitellaria maturation in response to male-derived cues.

In this study, we performed RNA interference (RNAi) screening of BATT–up-regulated genes in females at early stages postpairing, as identified by transcriptomic analysis. We discovered a transcription factor essential for the maturation of both the ovary and vitellaria. This transcription factor, encoded by a gene with two isoforms, acts specifically through its longer isoform, *znf362.1*, which is required for female sexual development. Using single-cell RNA sequencing (scRNA-seq), we characterized the dynamic expression profile of *znf362.1* at high resolution. Integrated multiomics and functional analyses revealed that ZNF362.1 directly regulated the transcription of the gonad-specific gene *cpeb1*, which in turn promoted meiosis resumption in the ovary by modulating polyadenylation of cyclin B1 mRNA and drives S_1_ cell differentiation in the vitellaria. These findings provide original insights into the molecular mechanisms governing female sexual maturation in schistosomes and elucidate a critical regulatory pathway linking male-derived signals to coordinated organ development. Understanding the core transcriptional networks that control parasite reproduction offers a potential foundation for developing strategies in schistosomiasis control.

## RESULTS

### Male-derived pheromone BATT stimulates expression of the transcription factor *znf362* to initiate female sexual development

**Fig. 1.**
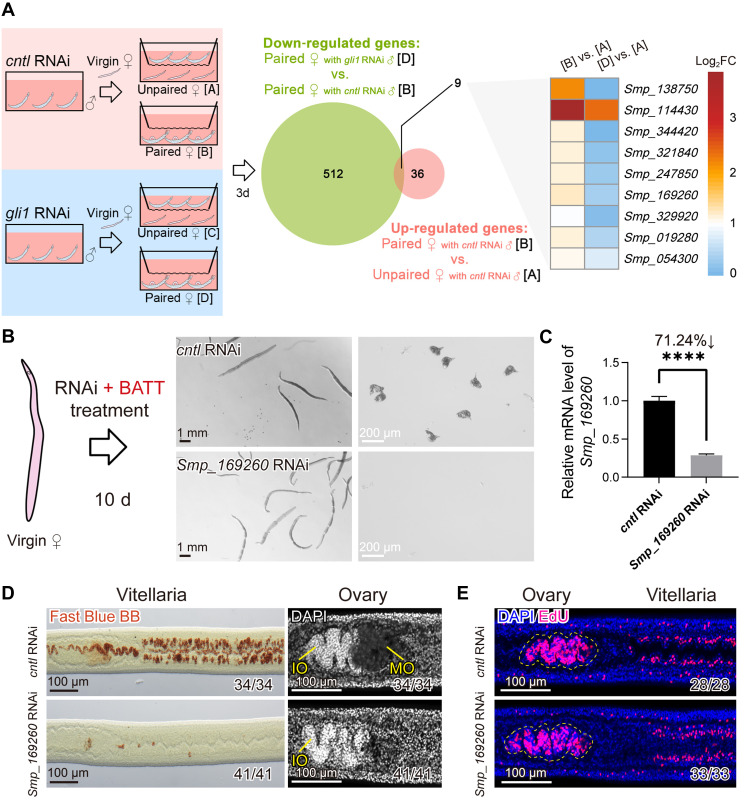
BATT-induced *znf362* regulates sexual maturation in virgin female schistosomes. (**A**) RNA-seq analysis identified nine genes in virgin females whose expression were up-regulated in response to male pheromone stimulation at 3 days (d) postpairing. The [B] versus [A] comparison identifies genes in virgin females that become up-regulated after pairing with control males capable of producing BATT. In contrast, the [D] versus [A] comparison reveals genes up-regulated in virgin females after pairing with BATT-deficient males (*gli1*-knockdown males that have lost the ability to produce BATT). *n* = 3. (**B**) Light microscopy showing morphology and oviposition of virgin females following control or *Smp_169260*-RNAi treatment in vitro for 10 days. Left, diagram showing the dsRNA treatment process; middle, whole-worm morphology (scale bars, 1 mm); right, egg morphology (scale bars, 200 μm). *n* = 3. (**C**) qPCR detection for *Smp_169260* knockdown in virgin females after 10 days of RNAi treatment. *n* = 3. Data are presented as mean ± SD. ****P < 0.0001. (**D**) Fast Blue BB and DAPI staining of vitellaria and ovaries in control or *Smp_169260*-RNAi virgin females after 10 days of treatment. Mature vitellaria stained reddish-brown with Fast Blue BB (left). In the ovary (right), immature oocytes (IO) appear as compact, DAPI-intense regions, while mature oocytes (MO) are lightly stained with larger cell volume. *n* = 3. Scale bars, 100 μm. (**E**) EdU labeling of proliferative cells in the ovaries and vitellaria following 7 days of treatment with control or *Smp_169260* dsRNA. EdU-positive cells are shown in magenta; nuclei were counterstained with DAPI (blue). The ovaries are outlined with yellow dashed lines. *n* = 3. Scale bars, 100 μm. Unpaired Student’s *t* test was applied for (C).

Virgin female schistosomes initiate sexual organ development only after the male-derived pheromone BATT is detected during pairing. To pinpoint genes that act downstream of BATT in females, and that are not merely induced by physical pairing, we performed reanalysis for published transcriptomic data from virgin females examined 3 days after pairing with either control males or with *gli1*-RNAi males that can pair normally but cannot synthesize BATT (*7*) (Fig. 1A). In females paired with control males, 45 genes were significantly up-regulated compared with unpaired virgins {[B] versus [A]; Log_2_FC ≥ 1, adjusted *P* (*P*.adj) < 0.05} (tables S3 and S4). Among these, nine genes did not show induction in females paired with *gli1*-RNAi males relative to those paired with control males ([D] versus [B]; Log_2_FC < 0, *P*.adj < 0.05), suggesting strict dependence on BATT signaling (Fig. 1A). RNAi screening of the nine candidates revealed that knockdown of *Smp_169260* blocked male-pheromone-induced sexual maturation of virgins and subsequent oviposition (Fig. 1, B and C, and fig. S1). Fast Blue BB and 4′,6-diamidino-2-phenylindole (DAPI) staining showed well-developed vitellaria with differentiated vitellocytes and mature ovaries in control-RNAi females, whereas *Smp_169260*-silenced females remained immature (Fig. 1D). Hydrochloric carmine staining further demonstrated that there were no mature oocytes in ovaries of the females from *Smp_169260*-RNAi group (fig. S2). To exclude the possibility that the elimination of proliferative stem cells caused this phenotype, we performed EdU incorporation assays. After 7 days of RNAi, proliferative cells in *Smp_169260*-RNAi females were comparable to that of the controls (Fig. 1E), suggesting that *Smp_169260* controls stem cell differentiation rather than their maintenance.

*Smp_169260*, annotated as a “putative zinc finger protein” in the WormBase ParaSite database (https://parasite.wormbase.org/), encodes two transcript variants: *Smp_169260.1* and *Smp_169260.2*, with coding sequence lengths of 5919 and 4254 bp, respectively (fig. S3). The predicted protein isoforms share a C-terminal region comprising 1403 amino acids, but differ in their N termini. Specifically, Smp_169260.1 contains a longer unique N-terminal extension of 569 amino acids, whereas Smp_169260.2 has a minimal N-terminal region of only 14 amino acids. Both isoforms harbor an identical zinc finger domain of 161 amino acids. Protein BLAST analysis identified homologous proteins in several model organisms, including zinc finger protein 362 (ZNF362) in *Homo sapiens* and *Danio rerio*, Rotund in *Drosophila melanogaster*, and LIN-29 in *Caenorhabditis elegans*. Both Rotund and LIN-29 are homologs of mammalian ZNF362 and belong to the Krüppel C2H2-type zinc finger protein family (*17*–*20*). Furthermore, the zinc finger domains are highly conserved across these species (fig. S4). On the basis of these findings, we designated *Smp_169260* as *znf362* in schistosomes.

### The longest isoform *znf362.1* is required for BATT-induced sexual maturation in virgin schistosomes

**Fig. 2.**
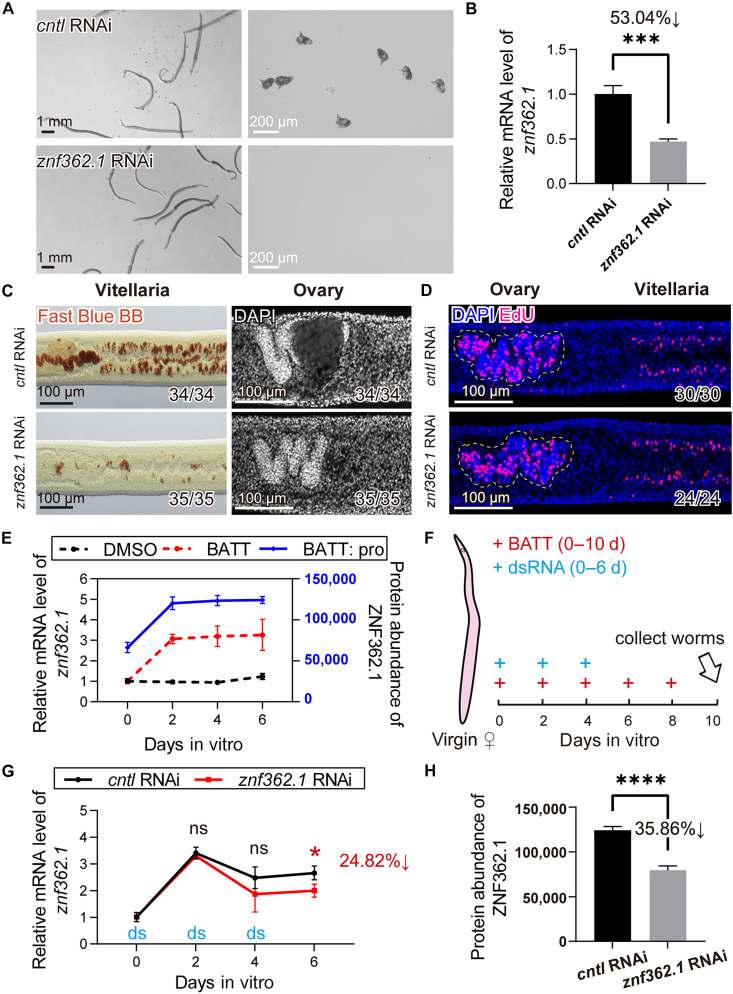
The longest transcript isoform *znf362.1* is essential for the reproductive development of virgin female schistosomes. (**A**) Light microscopy showing morphology and oviposition of virgin females after 10 days of in vitro treatment with control or *znf362.1* dsRNA. *n* = 3. Scale bars: whole-worm morphology, 1 mm; and egg morphology, 200 μm. (**B**) qPCR detection for *znf362.1* knockdown after 10 days of RNAi treatment. *n* = 3. Data are presented as mean ± SD. ***P < 0.001. (**C**) Fast Blue BB and DAPI staining of vitellaria and ovaries in control and *znf362.1*-RNAi virgin females after 10 days of treatment. Fast Blue BB staining of vitellaria (left); DAPI staining of ovaries (right). *n* = 3. Scale bars, 100 μm. (**D**) EdU labeling of proliferative cells in the ovaries and vitellaria following 7 days of treatment with control or *znf362.1* dsRNA. EdU-positive cells appear in magenta; nuclei are counterstained with DAPI (blue). The ovaries are outlined with yellow dashed lines. *n* = 3. Scale bars, 100 μm. (**E**) Quantification of *znf362.1* transcript and protein levels from D0 to D6 after BATT induction. Black and red dashed lines indicate transcript levels, while the blue solid line represents protein abundance. *n* = 3 to 4. Data are presented as mean ± SD. (**F**) Schematic diagram of the experimental design for 6-day RNAi treatment on female worms. (**G**) qPCR quantification of *znf362.1* transcript levels from D0 to D6 in control and RNAi-treated groups (*P = 0.03). *n* = 3. Data are presented as mean ± SD. (**H**) ZNF362.1 protein abundance at D6 in control and *znf362.1*-RNAi groups, as determined by DIA-based quantitative proteomics (****P < 0.0001). *n* = 4. Data are presented as mean ± SD. Unpaired Student’s *t* test was applied for [(B), (E), (G), and (H)].

Previously performed RNAi used a double-stranded RNA (dsRNA) fragment targeting both isoforms of *znf362*. To dissect the individual contributions of the two transcripts of *znf362* to female sexual development, we respectively synthesized isoform-specific dsRNAs targeting *znf362.1* and *znf362.2* (fig. S5A). As shown in Fig. 2 (A to C) and fig. S5 (B to D), knockdown of *znf362.1*, but not *znf362.2*, abolished sexual maturation and egg production in virgin females. Histological analyses using Fast Blue BB and DAPI staining revealed disrupted cellular differentiation in both the vitellaria and ovaries of *znf362.1*-RNAi females, phenocopying the defects we observed when both *znf362* isoforms were knockdown. Quantitative polymerase chain reaction (qPCR) confirmed that RNAi using isoform-specific dsRNA selectively reduced the target isoform without affecting the expression of the other isoform (fig. S5E). Moreover, EdU incorporation assays demonstrated that GSCs and S_1_ cells were still present in these tissues (Fig. 2D), indicating that *znf362.1* knockdown did not affect the maintenance of these proliferating cell populations.

Next, we examined the expression dynamics of *znf362.1* in virgin *S. mansoni* following BATT induction. qPCR analysis revealed a significant up-regulation of *znf362.1* mRNA, with a 3.05-fold increase at day 2 (D2) post-BATT treatment (*P* < 0.001), which remained elevated at D6 (3.25-fold versus D0, *P* < 0.01) (Fig. 2E and table S5). In contrast, control females without BATT exposure maintained low expression levels throughout the 0 to 6 day period. Proteomic sequencing confirmed a corresponding increase at the protein level: ZNF362.1 abundance rose by 81.45% at D2 (*P* < 0.0001) and remained high on D6, showing an 87.35% increase relative to D0 (*P* < 0.0001) (table S5). These findings indicate that *znf362.1* is rapidly and robustly induced in response to BATT stimulation.

To confirm that the early time window of sexual maturation (D0 to D6 after BATT induction) is critical, we performed *znf362.1* RNAi in a time window, and assessed the temporal dynamics of its transcript abundance. Virgin females were cultured in A169 medium supplemented with BATT every other day for a total of 10 days. We added *znf362.1*-specific dsRNA at D0, D2, and D4 (Fig. 2F). qPCR analysis revealed no significant difference in *znf362.1* mRNA expression between control and RNAi groups during the initial 4 days. By D6, RNAi significantly reduced *znf362.1* transcripts by 24.82% compared to that in controls (*P* = 0.03) (Fig. 2G and table S6). Consistently, RNAi decreased ZNF362.1 protein level by 35.86% at D6 post-RNAi (*P* < 0.0001) (Fig. 2H and table S6). Fast Blue BB and DAPI staining at D10 confirmed that this RNAi regimen effectively impaired sexual maturation in virgin females in the presence of BATT signaling (fig. S6).

### Single-cell profiling reveals *znf362.1* expression dynamics upon BATT induction

**Fig. 3.**
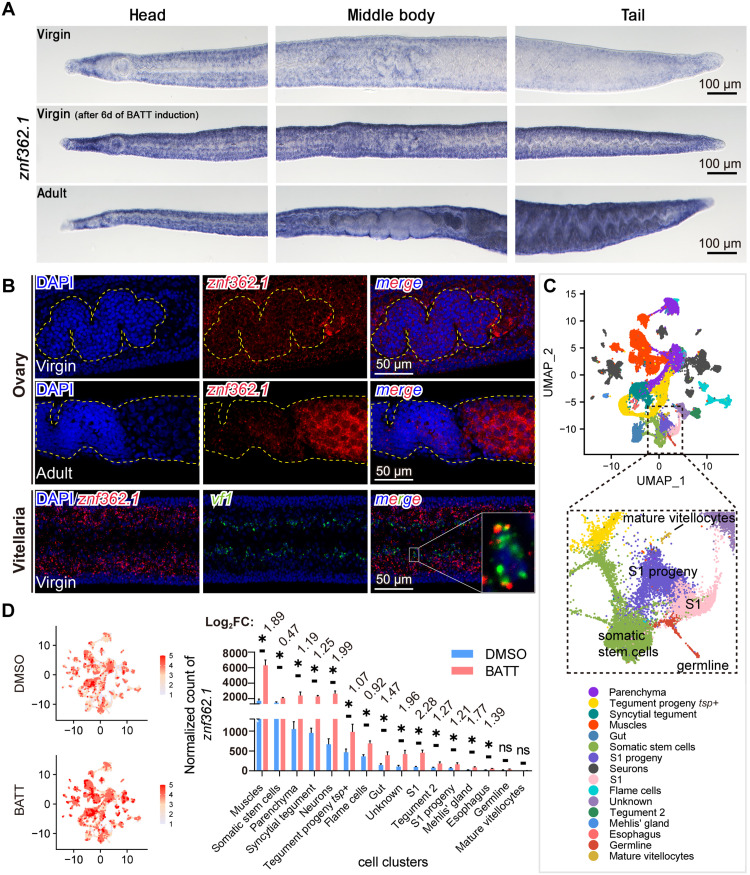
Expression profile of *znf362.1* and its single-cell dynamics in response to BATT stimulation. (**A**) WISH showing *znf362.1* expression in virgin females, virgin females treated with BATT in A169 for 6 days, and adult females. Purple staining indicates mRNA localization. Scale bars, 100 μm. Representative images of *n* > 12 parasites from three biological replicates. (**B**) FISH showing *znf362.1* expression in the ovaries and vitellaria of virgin females, and in the ovaries of adult females. Scale bars, 50 μm. Representative images of *n* > 12 parasites from three biological replicates. (**C**) UMAP plot of scRNA-seq data from virgin females, clustered on the basis of established cell type marker genes. (**D**) Single-cell resolution analysis of *znf362.1* expression in control and BATT-treated groups. Left: UMAP plots showing the relative expression level of *znf362.1*, with higher expression indicated by red. Right: Quantitative comparison of *znf362.1* expression across distinct cell types between control and BATT-treated groups at D2. *n* = 3. Data are presented as mean ± SD (*P.adj < 0.05).

Whole-mount in situ hybridization (WISH) revealed that *znf362.1* expressed broadly throughout virgin female schistosomes, with a potential enhancement in signal intensity following BATT treatment for 6 days (Fig. 3A). In adult females, the expression pattern of *znf362.1* was similar expression pattern to that observed in virgin stages. Given its essential role in female sexual development, fluorescence in situ hybridization (FISH) was further performed to detect its expression in reproductive organs. As shown in Fig. 3B, *znf362.1* expressed in the ovaries, with stronger signals detected in mature oocytes than in immature oocytes. In addition, *znf362.1* colocalized with *vitellogenic factor 1* (*vf1*), a specific marker for S_1_ cells in the vitellaria of virgin females. These results indicate that *znf362.1* expressed in both GSCs and S_1_ cells, albeit at relatively low abundance in virgin females.

To further resolve tissue-specific responses to BATT induction, we performed scRNA-seq on virgin females at D2 post-BATT or dimethyl sulfoxide (DMSO) treatment. Owing to the use of 3′-end scRNA-seq, which does not distinguish transcripts with shared 3′ regions, we considered potential contamination by *znf362.2*, which shares its 3′ coding sequence with *znf362.1*. To address this, we quantified the expression dynamics of *znf362.2* over the 0- to 6-day BATT induction period using qPCR. The data showed that *znf362.2* mRNA levels temporarily increased within 2 to 4 days (fig. S5F and table S7). The relative expression ratios of *znf362.1* to *znf362.2* were 6.69-, 12.18-, 12.31-, and 14.52-fold at 0, 2, 4, and 6 days postinduction, respectively. These findings indicate that *znf362.2* contributed less than 7.59% to the total *znf362* transcript pool following BATT treatment, confirming that the scRNA-seq data primarily reflect *znf362.1* expression. Therefore, we interpreted all the scRNA-seq signals annotated as *znf362* as *znf362.1* transcripts in subsequent analyses.

On the basis of established schistosome marker gene profiles (*21*), we annotated cells from these virgin females to 16 major clusters (Fig. 3C and table S8), including both somatic cell types, parenchyma, tegument, muscle, gut, neurons, flame cells, Mehlis’ gland, esophageal gland, and uncharacterized populations, and stem cell populations such as somatic stem cells (neoblasts), GSCs, and vitellarium stem cells (S_1_ cells). Notably, we detected a small population of mature vitellocytes (marked by *ataxin2/Smp_167830*) even in virgin females without BATT induction (fig. S7A). Although virgin females did not show mature oocytes (marked by *bmpg/Smp_078720*), we observed GSC progeny (marked by *meiob/Smp_333540*) under both conditions, suggesting a limited differentiation capacity in the absence of BATT. Quantitative assessment of cell type abundance further revealed that flame cells were the only population significantly reduced (by 29.72%) in response to BATT at D2 (fig. S7B). Comparative expression profiling showed that *znf362* transcripts significantly increased (*P* < 0.05) across nearly all cell types following BATT treatment, except in GSCs and mature vitellocytes (Fig. 3D and table S9). The most pronounced increase occurred in S_1_ cells within the vitellaria, highlighting this population as a primary site of *znf362.1* induction during early sexual development.

### Multiomics integration identifies *Smp_349410* as a key downstream target of ZNF362.1

**Fig. 4.**
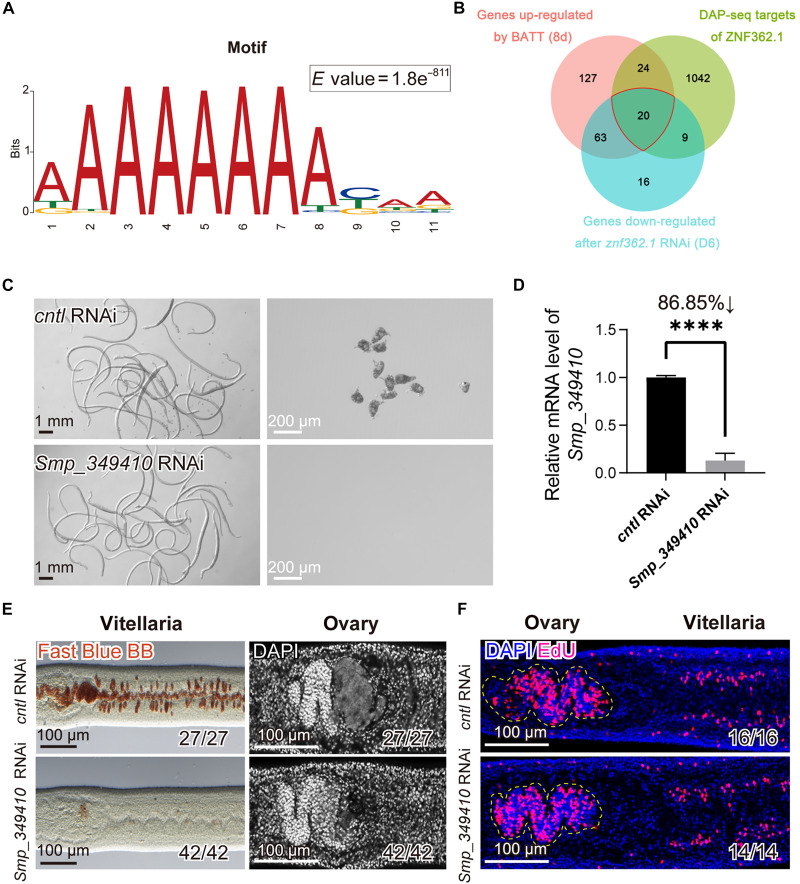
*Smp_349410* functions as a potential downstream target of ZNF362.1 in female sexual maturation. (**A**) Predicted DNA binding motif of ZNF362.1 with the highest probability, based on the DAP-seq findings. (**B**) Venn diagram showing the overlap among genes up-regulated after 8 days of BATT treatment, genes down-regulated after 6 days of *znf362.1* RNAi, and genes with promoter regions bound by the ZNF362.1 DBD as identified by DAP-seq. (**C**) Light microscopy showing morphology and oviposition of virgin females following 21 days of in vitro treatment with control or *Smp_349410* dsRNA (BATT inducing from D7). *n* = 3. Scale bars: whole-worm morphology, 1 mm; and egg morphology, 200 μm. (**D**) qPCR detection for *Smp_349410* knockdown in virgin females after 21 days of RNAi treatment (BATT inducing from D7). *n* = 3. Data are presented as mean ± SD. ****P < 0.0001. (**E**) Fast Blue BB and DAPI staining of vitellaria and ovaries in control and *Smp_349410*-RNAi virgin females after 21 days of treatment (BATT inducing from D7). Fast Blue BB staining of vitellaria (left); DAPI staining of ovaries (right). *n* = 3. Scale bars, 100 μm. (**F**) EdU labeling of proliferative cells in the ovaries and vitellaria following 7 days of treatment with control or *Smp_349410* dsRNA. EdU-positive cells appear in magenta; nuclei are counterstained with DAPI (blue). The ovaries are outlined with yellow dashed lines. *n* = 3. Scale bars, 100 μm. Unpaired Student’s *t* test was applied for (D).

Transcription factors modulate gene expression by binding to specific DNA motifs within the promoter regions of target genes. To elucidate the regulatory network governed by ZNF362.1 during female sexual maturation, we used DNA affinity purification sequencing (DAP-seq) to map genome-wide binding sites of the ZNF362.1 DNA binding domain (DBD) in females. Peak-calling analysis revealed that ZNF362.1 preferentially binds to intronic regions (40.56%) and distal intergenic regions (37.80%) of the genome, with 9.65% of peaks located within promoter regions (fig. S8, A and B, and table S10). Motif discovery analysis identified seven enriched sequence motifs (*E* value ≤0.05), among which the hexamer “AAAAAA” showed the highest enrichment (*E* value = 1.8 × 10^−811^) and a centrally enriched, unimodal distribution within ZNF362.1-bound peaks (Fig. 4A and fig. S8C).

To identify potential downstream genes regulated by ZNF362.1, we conducted RNA-seq after *znf362.1* RNAi. After 6 days of treatment, RNAi significantly down-regulated 108 genes (Fig. 4B, fig. S9, and table S11), of which BATT induction up-regulated 83 genes (table S12). Of the 83 genes, 20 contained the canonical ZNF362.1 binding motif within their promoter regions, suggesting they are direct transcriptional targets (table S13). Cross-referencing with scRNA-seq data and the SchistoCyte Atlas (https://collinslab.org/schistocyte/), we excluded 10 genes due to their restricted expression in mature vitellocytes, a cell type in which *znf362.1* was not up-regulated following BATT induction (fig. S10). The remaining 10 candidate genes expressed in S_1_ cells and/or oocytes. Functional screening via RNAi, using the same protocol as for *znf362.1*, revealed that knockdown of *Smp_349410* resulted in complete failure of vitellaria maturation, while ovarian development remained unaffected (fig. S11). qPCR confirmed a knockdown efficiency of 52.94% (*P* = 0.04) (fig. S11B). To enhance silencing efficacy, we extended the RNAi treatment by an additional 7 days before BATT induction, which improved knockdown efficiency to 86.85% (Fig. 4, C and D). Under these conditions, both vitellaria and ovaries failed to mature in *Smp_349410*-RNAi females, whereas control and other RNAi groups presented fully developed gonads and egg production (Fig. 4E and fig. S12). Similar to *znf362.1* RNAi, *Smp_349410* knockdown did not affect the maintenance of GSCs or S_1_ cells (Fig. 4F).

### ZNF362.1 directly activates *Smp_349410* transcription through promoter binding

**Fig. 5.**
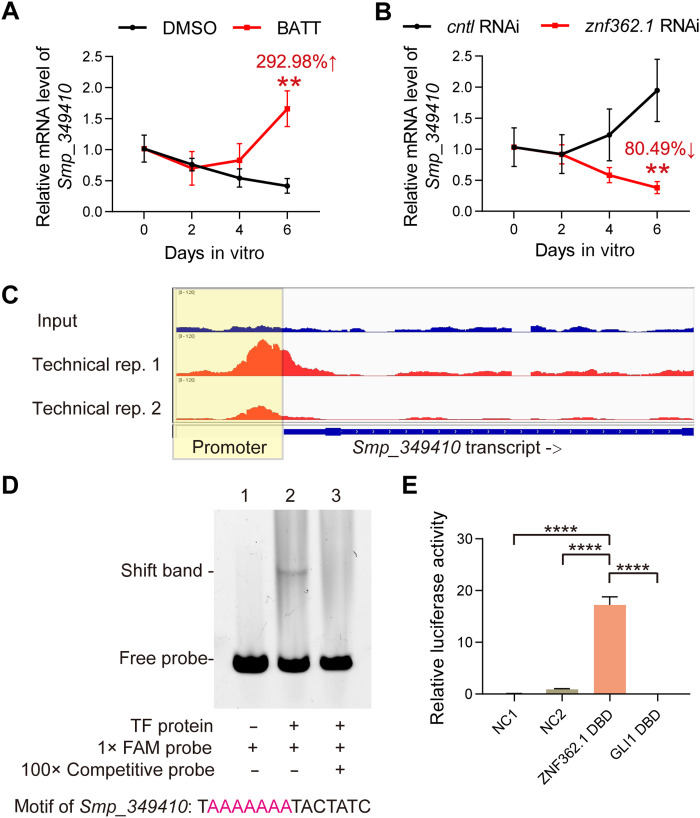
*Smp_349410* is a direct transcriptional target of schistosome ZNF362.1. (**A**) Quantification of *Smp_349410* transcript levels by qPCR in virgin females from D0 to D6 under control or BATT-induced conditions. *n* = 3. Data are presented as mean ± SD. **P < 0.01. (**B**) Quantification of *Smp_349410* transcript levels by qPCR in virgin females during 0 to 6 days in control or *Smp_349410*-RNAi groups under BATT induction. *n* = 3. Data are presented as mean ± SD. **P < 0.01. (**C**) Integrated Genomics Viewer (IGV) visualization of DAP-seq coverage at the *Smp_349410* locus. Tracks show uniquely mapped reads aligned to the *S. mansoni* reference genome (version 10) from two technical replicates and one input control. The *y* axis indicates per-base read coverage. The promoter region was highlighted with a yellow box. (**D**) EMSA validating ZNF362.1 binding to the *Smp_349410* promoter motif. Lane 1: FAM-labeled probe only (no protein); Lane 2: FAM-labeled probe with ZNF362.1 DNA binding domain (DBD); lane 3: competition with 100-fold molar excess of unlabeled probe. (**E**) Dual-luciferase reporter assay validating the transcriptional activation of the *Smp_349410* promoter by the ZNF362.1 DBD. NC1 (negative control 1): Empty pcDNA3.1 vector + reporter plasmid containing the *Smp_349410* promoter. NC2 (negative control 2): ZNF362.1 DBD expression plasmid + reporter lacking the *Smp_349410* promoter. ZNF362.1 DBD: ZNF362.1 DBD-VP64-NLS expression plasmid + reporter containing the *Smp_349410* promoter. GLI1 DBD: GLI1 DBD-VP64-NLS expression plasmid + reporter containing the *Smp_349410* promoter. *n* = 3. Data are presented as mean ± SD. ****P < 0.0001. Unpaired Student’s *t* test was applied for [(A), (B), and (E)].

To characterize the expression dynamics of *Smp_349410*, we measured its mRNA level over a 6-day period following BATT stimulation. As shown in Fig. 5A and table S14, its expression progressively increased in BATT-treated females, with a 292.98% elevation at D6 compared to that of the DMSO controls (*P* < 0.01). In contrast, *znf362.1* knockdown abrogated this BATT-induced up-regulation (Fig. 5B and table S15) (*P* < 0.01), suggesting that *Smp_349410* is transcriptionally regulated by ZNF362.1.

The Integrative Genomics Viewer (IGV) plot of DAP-seq revealed that the ZNF362.1 DBD enriching on promoter of *Smp_349410* (Fig. 5C). To validate whether ZNF362.1 directly regulates *Smp_349410*, we expressed the DBD of ZNF362.1 (residues 561–741) in *Escherichia coli* and performed electrophoretic mobility shift assay (EMSA) (fig. S13). Incubation of the recombinant ZNF362.1 DBD with a labeled probe containing the target motif from the *Smp_349410* promoter resulted in a distinct shift band (Fig. 5D). Competition with a 100-fold excess of unlabeled probe abolished this shift, confirming the specific binding of ZNF362.1 to the promoter motif. To further assess the transcriptional regulation under physiological conditions, we conducted dual-luciferase reporter assays (fig. S14). Cotransfection of pcDNA3.1-His-Halo-ZNF362.1 DBD-VP64-NLS with pGL4.23-*Smp_349410*_promoter significantly enhanced luciferase activity by 193.86-fold and 19.22-fold relative to that of two negative controls (NC1: empty expression vector; and NC2: empty reporter vector) (*P* < 0.0001) (Fig. 5E). In contrast, a nonspecific DBD from GLI1 had no effect on *Smp_349410* promoter activity. Collectively, these data demonstrate that ZNF362.1 has potency to directly regulate *Smp_349410* transcription by binding to its promoter.

### *Smp_349410* is restricted to the ovaries and vitellaria of female parasites

**Fig. 6.**
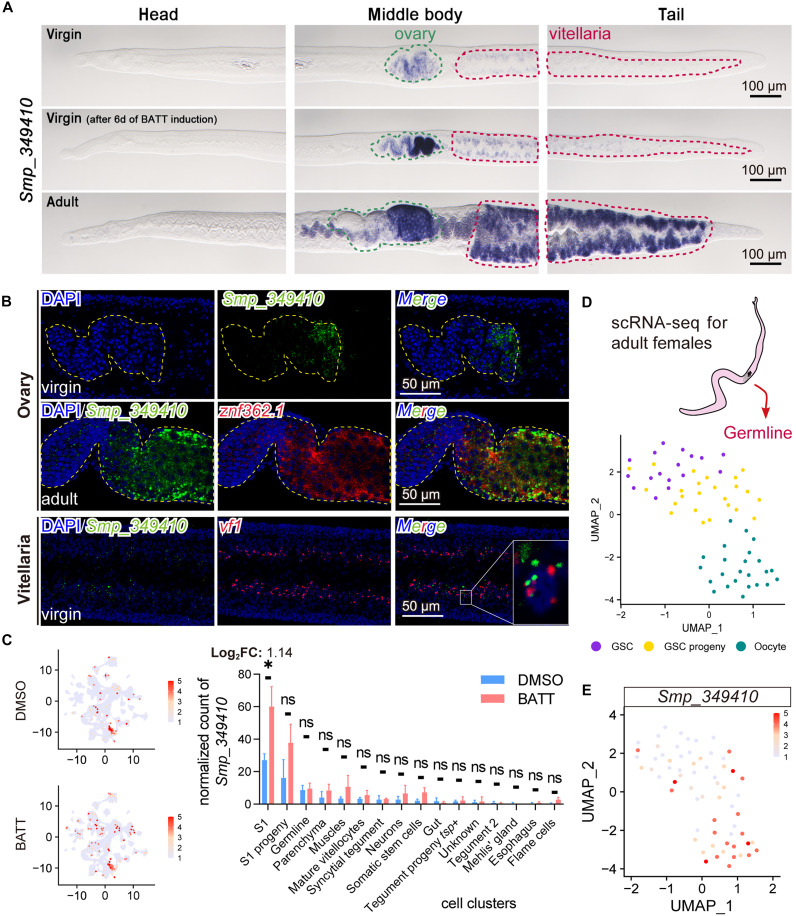
*Smp_349410* is specifically expressed in the gonads of female schistosomes. (**A**) WISH showing *Smp_349410* expression in virgin females, virgin females treated with BATT in A169 for 6 days, and adult females. Purple staining indicates mRNA localization. Scale bars, 100 μm. Representative images of *n* > 12 parasites from three biological replicates. (**B**) FISH showing *Smp_349410* expression in the ovaries and vitellaria of virgin females, and in the ovaries of adult females. Scale bars, 50 μm. Representative images of *n* > 12 parasites from three biological replicates. (**C**) Single-cell resolution analysis of *Smp_349410* expression in control and BATT-treated groups. Left: UMAP plots showing the average expression level of *Smp_349410*, with higher expression indicated in red. Right: Quantitative comparison of *Smp_349410* expression across distinct cell types between control and BATT-treated groups at D2. *n* = 3. Data are presented as mean ± SD (*P.adj < 0.05). (**D**) UMAP plot of germline scRNA-seq data from adult females, clustered on the basis of established cell type marker genes. (**E**) UMAP plot of *Smp_349410* expression in the germline of adult females, with higher expression indicated in red.

WISH revealed that both virgin and adult female worms exclusively expressed *Smp_349410* in the ovaries and vitellaria (Fig. 6A), but males rarely expressed it (fig. S15). Upon 6-day BATT induction, the WISH signal appeared to markedly enhance in these reproductive tissues, particularly in the posterior region of the ovaries, where mature oocytes are localized. To further resolve its cell type–specific expression, we conducted FISH. As shown in Fig. 6B, *Smp_349410* exhibited strong expression in the posterior ovaries of virgin females, corresponding to regions enriched in differentiated oocytes, whereas GSC population displayed low expression. We further corroborated this spatial expression pattern in adult females, where a robust FISH signal appeared in oocyte-dominated regions of the ovaries. In vitellaria, *Smp_349410* signal colocalized with the S_1_ cell marker *vf1* (Fig. 6B), and scRNA-seq data further confirmed its coexpression with *znf362.1* (fig. S16). Thus, *Smp_349410* colocalized with its direct transcriptional regulator *znf362.1* in both the ovary and vitellaria, suggesting coordinated regulation within these reproductive tissues.

To assess its BATT responsiveness in these specific cell populations, we analyzed scRNA-seq data. In virgin females (DMSO group), *Smp_349410* localized primarily to S_1_ cells within the vitellaria, and BATT treatment for 2 days significantly up-regulated its expression (Log_2_FC = 1.14, *P*.adj < 0.05; Fig. 6C). Given the absence of mature oocytes in virgin females, we further investigated *Smp_349410* expression in adult females using scRNA-seq (Fig. 6D and fig. S17). This analysis confirmed that *Smp_349410* expression progressively increases across cell stages during the maturation of both the ovary and vitellaria. (Fig. 6E and fig. S17C). Collectively, these data suggest that female reproductive organs exclusively expressed *Smp_349410*, which exhibits comparable up-regulation in the ovary and vitellaria during differentiation.

### CPEB1-mediated ovary development via regulation of cyclin B1 mRNA polyadenylation

*Smp_349410* is annotated as an “RRM domain-containing protein” in the WormBase ParaSite database. This gene encodes a single transcript with a coding sequence (CDS) of 2937 bp, producing a protein of 978 amino acids. Conserved domain (CD) database analysis revealed that the protein contains two RNA recognition motifs (RRMs) and a ZZ-type zinc finger domain at the C terminus (fig. S18A). Phylogenetic analysis showed that Smp_349410 is most closely related to *Schmidtea mediterranea* CPEB1 and clusters with *C. elegans* CPEB3, *D. melanogaster* Orb, *D. rerio* CPEB1, and *H. sapiens* CPEB1 (fig. S18B and table S16). Furthermore, the C-terminal region is highly conserved across these species (fig. S19). These findings support the conclusion that *Smp_349410* encodes a homolog of CPEB1, a classical post-transcriptional regulator that binds to RNA in a sequence-specific manner to modulate cytoplasmic polyadenylation and translation initiation during oocyte maturation, early embryonic development, and synaptic function in neurons (*22*–*25*).

CPEB1 is a well-characterized RNA binding protein in mammals that uses its RRM domains for RNA recognition. Homology modeling between the RRM domains of *Sm*CPEB1 and human CPEB1 yielded a root mean square deviation value of 3.85 Å (fig. S18C). Since this value exceeds the conventional similarity threshold of 3.0 Å, it indicates notable structural divergence in the RNA binding domains of *Sm*CPEB1, suggesting that the RNA target specificities of *Sm*CPEB1 are distinct from those of its mammalian counterparts.

**Fig. 7.**
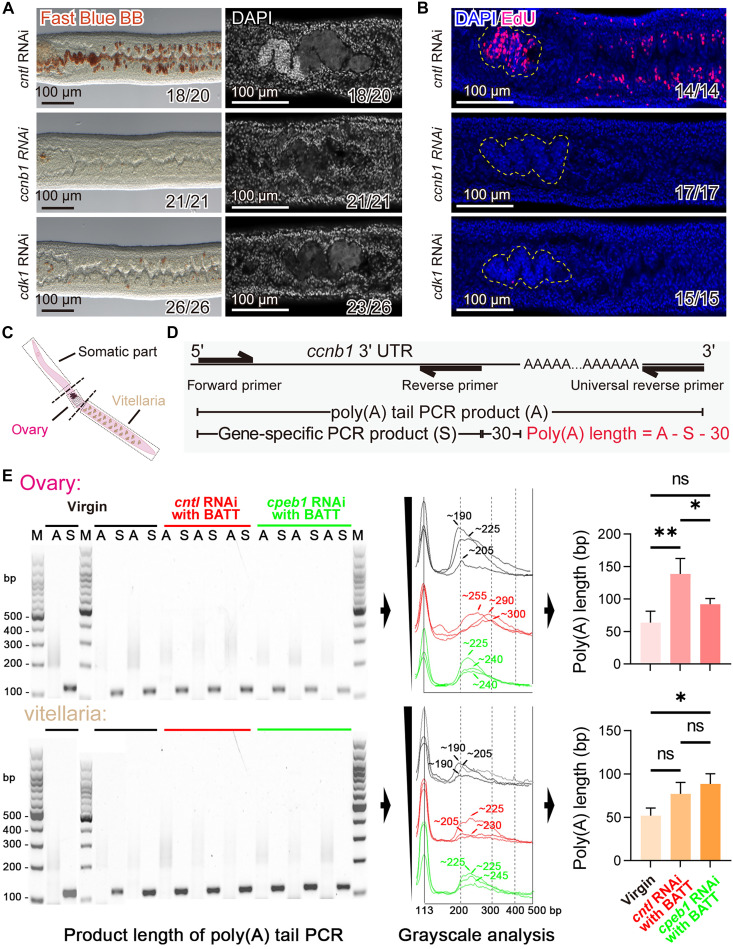
*Cpeb1* regulates cyclin B1 mRNA polyadenylation in female schistosomes during reproductive development. (**A**) Fast Blue BB and DAPI staining of vitellaria and ovaries in females treated with control, *ccnb1* or *cdk1* dsRNA after 19 days (BATT inducing from D7). Fast Blue BB staining of vitellaria (left); DAPI staining of ovaries (right). *n* = 3. Scale bars, 100 μm. (**B**) EdU labeling of proliferative cells in the ovaries and vitellaria following 7 days of treatment with control, *ccnb1*, or *cdk1* dsRNA. EdU-positive cells appear in magenta; nuclei are counterstained with DAPI (blue). The ovaries are outlined with yellow dashed lines. *n* = 3. Scale bars, 100 μm. (**C**) Schematic illustration of somatic tissue, ovary and vitellaria dissection for RNA extraction; boxed regions excised from female worms. (**D**) Schematic illustration of the PCR-based assay for analyzing cyclin B1 mRNA poly(A) tail length. Poly(A) PCR amplifies the poly(A) region (A); gene-specific PCR targets the 3′UTR (S) for alignment. Tail length calculated as A − S − 30 [S = 113 bp; 30 = distance from reverse primer to poly(A) start]. (**E**) Poly(A) tail analysis of cyclin B1 mRNA across somatic tissue, ovary, and vitellaria in virgin, BATT-induced control RNAi, and BATT-induced *cpeb1* RNAi females. A, poly(A) tail PCR product (A); S, gene-specific PCR product (S); M, 100 bp DNA ladder. Relative poly(A) tail length was estimated from peak summits (arrows) after grayscale analysis. *n* = 3. Data are presented as mean ± SD. **P < 0.01 and *P < 0.05. Unpaired Student’s *t* test was applied for (E).

In model organisms, CPEB activates the translation of target mRNAs through polyadenylation, with classic mRNA targets including *ccnb1* (encoding cyclin B1) and *Mos* (*23*, *25*). In schistosomes, we identified only a homolog of cyclin B1 (Smp_082490). Cyclin B1 is a key regulator of cell cycle progression, forming a complex with CDK1 to mediate the G_2_/M transition during mitosis and meiosis resumption (*26*). To assess its function in schistosomes, we performed RNAi targeting *ccnb1* and *cdk1* (*Smp_080730*). Knockdown of either gene resulted in complete failure of ovary and vitellaria maturation, characterized by the loss of all proliferative cells (Fig. 7, A and B). Similarly, treatment with the CDK1-specific inhibitor RO-3306 recapitulated this phenotype, suppressing sexual maturation and GSC maintenance (fig. S20). These results support the conserved role of the cyclin B1/CDK1 complex in cell cycle regulation in schistosomes.

To investigate whether *Sm*CPEB1 regulates cyclin B1 mRNA polyadenylation, we measured the poly(A) tail length following *cpeb1* RNAi in the ovaries and vitellaria (Fig. 7, C to E). We controlled for potential background signals from somatic cells (e.g., tegument, parenchyma, muscle, and neurons) by PCR product smears from anterior body segments lacking sexual organs and analysis of gray value intensity profiles (Fig. 7C and fig. S21). We detected no polyadenylation change in the somatic cells of virgin or control RNAi worms. In the ovary, the mean poly(A) tail length of cyclin B1 mRNA increased from 63.67 ± 17.56 bp in virgins to 138.7 ± 23.63 bp in sexually mature worms (*P* = 0.0049). The *cpeb1* knockdown significantly reduced this length to 92.00 ± 8.66 bp (*P* = 0.0412) (Fig. 7E). In contrast, in the vitellaria, the poly(A) tail length did not significantly increase in sexually mature worms (*P* = 0.0783), and *cpeb1* knockdown did not affect it (88.67 ± 11.55 bp; *P* = 0.3139) (Fig. 7E). These results indicate that CPEB1 promotes oocyte maturation in the ovary by regulating cyclin B1 mRNA polyadenylation. In vitellaria, however, CPEB1 appears to regulate sexual development through alternative regulatory pathways that are independent of cyclin B1 mRNA polyadenylation.

**Fig. 8.**
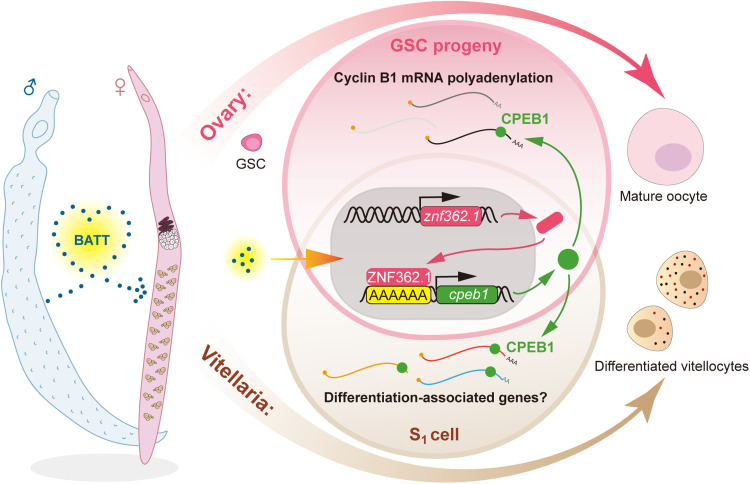
Schematic diagram illustrating the ZNF362.1-mediated transcriptional regulation of sexual development in female *S. mansoni* in response to male-derived BATT stimulus. In virgin females, differentiation of the ovary and vitellaria remains arrested, with development stalled at the GSC progeny stage in the ovary and at the S_1_ cell stage in the vitellaria. Upon pairing, the male-derived pheromone BATT induces the up-regulation of the transcription factor *znf362.1* in females. ZNF362.1 subsequently activates *cpeb1* transcription in both the ovary and vitellaria. In the ovary, elevated CPEB1 levels enhance the polyadenylation of cyclin B1 mRNA, a key regulator of meiosis, thereby promoting oocyte maturation. In the vitellaria, CPEB1 influences the differentiation of proliferative S_1_ cells through a pathway that appears independent of cyclin B1 mRNA polyadenylation.

Together, our findings indicate that the transcription factor ZNF362.1 in female schistosomes up-regulates in response to the male-derived pheromone BATT. ZNF362.1 binds to a specific DNA motif to activate transcription of the downstream gene *cpeb1*. Elevated CPEB1 levels, in turn, direct the maturation of proliferative cells in both the ovary and vitellaria. Notably, CPEB1 appears to regulate ovary and vitellaria maturation through distinct molecular mechanisms (Fig. 8).

## DISCUSSION

In this study, we identified the BATT-responsive transcription factor ZNF362 as a central regulator of ovary and vitellaria maturation in female schistosomes. It encodes two transcript isoforms, *znf362.1* and *znf362.2*, which generate proteins of different lengths but share a conserved DBD. Functional analyses revealed that only the long isoform, *znf362.1*, is indispensable for female sexual development. Isoform-specific functions of ZNF362 homologs have also been reported in other species, underscoring the evolutionary importance of transcript divergence. In *D. melanogaster*, the long *rotund* isoform is required for appendage development, whereas the shorter *roughened eye* isoform regulates ommatidial organization and photoreceptor number (*17*, *18*, *27*–*29*). Similarly, in *C. elegans*, distinct *lin-29* isoforms coordinate seam cell cycle exit and fusion during the L4-to-adult transition, while differentially controlling alae formation (*19*, *20*). Together, these examples highlight isoform specialization as a conserved strategy for achieving spatially and temporally distinct developmental outcomes. Compared with homologs in *Drosophila* and *C. elegans* (typically <950 amino acids), schistosome ZNF362 has an unusually elongated low-complexity domain (LCD), largely overlapping with intrinsically disordered regions. LCD extensions are known to broaden the range of protein-protein interactions, promote biomolecular phase separation, and facilitate the dynamic assembly of transcriptional complexes (*30*–*32*). The expansion of LCDs in transcriptional regulators is thought to enhance both the flexibility and amplitude of gene regulatory responses (*33*, *34*). We propose that the extended LCD of schistosome ZNF362 represents an evolutionary innovation that enables the integration of species-specific external cues, such as the male-derived pheromone BATT, into robust transcriptional outputs. Notably, several genes other than *znf362* are also responsive to BATT, including *Smp_114430*, which shows strong up-regulation. However, RNAi-mediated knockdown of these genes did not result in defects in female reproductive development or subsequent egg production, suggesting that they may function in other tissues or biological pathways.

At the single-cell level, 2 days after BATT stimulation, most cell types in virgin females showed no significant numerical changes, except for a reduction in flame cells. As components of the excretory system (*35*), this reduction may reflect an adjustment associated with metabolic shifts. The numbers of germline and vitelline cells at different differentiation stages remained unchanged, indicating that gonadal differentiation had not yet begun at this early time point. Nevertheless, *znf362.1* expression was significantly up-regulated across multiple somatic and reproductive cell types. Given its broad expression, ZNF362.1 may function in both reproductive and nonreproductive tissues. However, we did not detect apparent somatic defects in *znf362.1* RNAi females, and multiomics analyses did not identify somatic-cell–specific downstream targets during gonadal maturation. In contrast, we found the downstream target *cpeb1* (*Smp_349410*) exclusively expressed in the ovary and vitellaria and it is required for reproductive development. These data suggest that ZNF362.1 primarily governs sexual development through *cpeb1*, although its potential somatic functions merit further examination.

The ovary and vitellaria are the two principal reproductive organs of female schistosomes. In virgin females, the undifferentiated cells within these organs, GSCs in the ovary and S_1_ vitelline cells in the vitellaria, are self-proliferative but remain incapable of differentiating into mature cells without stimulation from a male partner (*21*, *36*, *37*). Nevertheless, occasional mature vitelline cells have been observed in virgin females (*38*, *39*), and are detectable by Fast Blue BB staining even in the absence of male induction. Consistent with this, our single-cell atlas of virgin females (DMSO-treated controls) revealed small populations of S_1_ progeny and mature vitellocytes in the vitellaria, as well as GSC progeny in the ovary, indicating that limited self-activated differentiation can occur in these organs. However, compared with the corresponding differentiated populations in adult females, these cells exhibited only a restricted set of marker genes, suggesting that autodifferentiated cells in virgin females represent aberrant or incomplete maturation states.

Although the ovary and vitellaria are both classified as female reproductive organs, they represent distinct tissues with divergent developmental trajectories. The ovary consists of germline cells, in which GSCs undergo meiosis to generate oocytes, whereas the vitellaria are composed of specialized somatic cells, with vitellocytes differentiating from proliferative S_1_ cells to provide eggshell components and nutrients for embryogenesis. *znf362.1* displayed differential expression patterns between these organs. In the ovary, its expression was low in GSCs and not responsive to BATT stimulation at early stages (D2), but increased in differentiating oocytes. In contrast, in the vitellaria, *znf362.1* expression markedly up-regulated in proliferative S_1_ cells after 2 days of BATT treatment. These findings indicate that *znf362.1* regulates the maturation of the ovary and vitellaria through heterogeneous mechanisms. Despite these differences, both organs share the same downstream effector of *znf362.1*, *cpeb1* (*Smp_349410*), which specifically expresses in the ovary and vitellaria. Notably, this gene was absent from earlier *S. mansoni* genome assemblies and only incorporated in the recently improved reference genome (version 10, publicly released in WormBase ParaSite, https://parasite.wormbase.org/Schistosoma_mansoni_prjea36577/Info/Index/) (*40*–*42*). CPEB1 plays a conserved role in oogenesis across diverse species, including *S. mediterranea* (*43*), *Xenopus* (*44*), *Drosophila* (*45*), clams (*46*), zebrafish (*47*), and mice (*48*), suggesting that its role in regulating gonadal maturation is widely conserved throughout the animal kingdom. In *Xenopus* and mice, CPEB1 represses the translation of maternal cyclin B1 mRNA (encoded by the *ccnb1* gene) by binding the maturation-type cytoplasmic polyadenylation element (mCPE) in its 3′ untranslated region (UTR) in an unphosphorylated state. Upon meiotic entry, CPEB1 phosphorylation triggers polyadenylation and translational activation of cyclin B1, thereby coordinating the temporal progression of oocyte maturation (*24*, *26*, *49*, *50*). Consistent with this conserved role, we found that *ccnb1* and *cdk1* are critical for maintaining stem cell cycling in schistosomes, and poly(A) tail assays demonstrated that *cpeb1* knockdown shortened the poly(A) tails of cyclin B1 mRNA in the ovary, but not in vitellaria. Although cell type–specific knockdown of *ccnb1* was not feasible, the oocyte-specific expression of *cpeb1*, together with its regulation of cyclin B1 mRNA, strongly supports its role as a key modulator of oocyte cell cycle progression in schistosomes. More specifically, these findings suggest that CPEB1 regulates the translation of cyclin B1, thereby mediating the G_2_/M transition and ultimately promoting the resumption of meiosis in oocytes. In the vitellaria, however, S_1_ cells predominantly expressed *cpeb1* and up-regulated its expression by BATT in virgin females, but *cpeb1* knockdown did not affect cyclin B1 polyadenylation. This suggests that CPEB1 performs a distinct, cyclin B1–independent function in vitellaria maturation, a role that warrants further investigation.

This study identified *znf362.1* as a “switch transcription factor” that initiates sexual maturation in female schistosomes. However, the precise mechanism by which BATT induces *znf362.1* transcription remains unresolved. BATT functions as a pheromone conserved in both schistosomes and hermaphroditic planarians (*51*). As a dipeptide, BATT falls within the class of peptide/protein hormones (e.g., insulin, growth hormone, and adrenocorticotropic hormone), which are water soluble and therefore unable to diffuse across the plasma membrane (*52*–*54*). Instead, such hormones typically act through cell-surface receptors, most commonly G protein–coupled receptors or enzyme-linked receptors, that activate intracellular signaling cascades via second messengers such as adenosine 3′,5′-monophosphate, IP_3_/diacylglycerol, or calcium-calmodulin. Given the widespread up-regulation of *znf362.1* observed following BATT stimulation, it is plausible that a broadly expressed membrane receptor mediates the recognition and transduction of the BATT signal, thereby triggering downstream pathways that culminate in transcriptional activation.

In summary, this study demonstrated that *znf362.1* mediates female sexual development in schistosomes. Upon pairing with males, the male-derived pheromone BATT induces the up-regulation of *znf362.1* in virgin females. In the ovary, elevated ZNF362.1 binds to the promoter of *cpeb1*, activating its transcription. CPEB1 in turn regulates the translation of meiosis-related target mRNAs, most likely cyclin B1, thereby ensuring the proper temporal progression of meiosis (Fig. 8). In the vitellaria, however, the mechanism differs: BATT-induced *znf362.1* up-regulation in proliferative S_1_ cells activates *cpeb1*, which promotes S_1_ cell differentiation through a pathway independent of cyclin B1. Together, these findings reveal a finely tuned regulatory system in which a common BATT signal orchestrates the maturation of two distinct reproductive organs via shared upstream regulators but divergent downstream pathways. This work not only advances our understanding of flatworm reproductive biology but also highlights potential molecular targets for the control of schistosomiasis.

One limitation of this study is the use of a single-animal model, which may not fully represent the diversity of responses in other *Schistosoma* species. However, by mining published transcriptomic and proteomic datasets, we observed that *znf362* and *cpeb1* are also up-regulated in females after pairing with male worms in vivo in *Schistosoma japonicum* (*55*–*57*), supporting the existence of a conserved, BATT-triggered regulatory network in schistosomes. Another potential limitation is that our study focuses primarily on the BATT-mediated pathway, whereas the regulatory network induced by natural pairing is likely more complex. In particular, the broader effects of physical pairing on female physiology, beyond reproductive system development, remain to be explored in future studies. In addition, we identified mRNA target of *Sm*CPEB1 in this study through literature mining, rather than systematic screening, and there may be other genes involved in the regulation of female reproductive development.

## MATERIALS AND METHODS

### Parasites

Miracidia were released from eggs obtained from livers of infected female BALB/c mice and used to infect the intermediate host *Biomphalaria glabrata*. To obtain sexually mature male and female worms, each snail was exposed to 5 to 10 miracidia for mixed infection. To obtain immature virgin females, each snail was exposed to a single miracidium. For snails infected with a single miracidium, each snail was maintained individually and 5 to 10 cercariae released from each snail were collected for PCR-based sex identification (*58*). Snails proven to release female cercariae were kept individually for subsequent infection. Mice were infected with only female or both genders of *S. mansoni* cercariae (Puerto Rico strain) by tail exposure. Virgins and adult worms (6 to 7 weeks postinfection) were harvested from infected mice by hepatic portal vein perfusion with ice-chilled 0.9% (w/v) NaCl and 0.005% (w/v) heparin. Worms used in vitro culture were rinsed with BM169 and cultured in A169 medium (BM169 medium supplemented with 200 μM ascorbic acid) containing 10% fetal bovine serum and 1× antibiotic-antimycotic (*15*, *59*, *60*). The worms of biological replicates in this study were defined as worms collected from different mice. All experiments involving vertebrate animals were performed in accordance with protocols approved by the Institutional Animal Care and Use Committee of Fudan University (APN: 2021JS0078).

### RNA interference

dsRNA production and RNAi treatment were performed as previously described (*37*, *61*). Oligo sequences used to construct pJC53.2 plasmids for dsRNA templates are listed in table S1. A dsRNA derived from two bacterial genes was used as a negative control for all RNAi experiments (*61*). D0 was defined as the first day of the experiment. In the RNAi screening for BATT–up-regulated genes, virgin females were treated on D0 with dsRNA (30 μg/ml) and 50 μM BATT in A169 medium. Both dsRNA and BATT were replenished every 2 days until D10, when phenotypes were evaluated. In the RNAi screening for *znf362.1*–up-regulated genes, virgin females were subjected to RNAi for 7 days in BM169 medium with dsRNA (30 μg/ml), followed by continued RNAi in A169 medium supplemented with dsRNA (30 μg/ml) and 50 μM BATT for an additional 10 days.

### Parasite staining and imaging

Parasites were anesthetized in 0.25% tricaine dissolved in BM169 medium, euthanized in 0.6 M MgCl_2_, and subsequently fixed for 4 hours in 4% paraformaldehyde with 0.3% Triton X-100. WISH, FISH (*37*, *62*), Fast Blue BB, DAPI, EdU labeling (*15*), and hydrochloric carmine staining (*9*, *63*) were performed as previously described. For in situ hybridization, riboprobes were synthesized from pJC53.2 plasmids constructed using primers listed in table S1. For Fast Blue BB staining, worms were incubated in freshly prepared 1% Fast Blue BB solution (Sigma-Aldrich, USA; catalog no. 44670) in PBSTx (phosphate-buffered saline + 0.3% Triton X-100) for 10 min, followed by incubation with DAPI (1 μg/ml) in PBSTx overnight. For EdU labeling, worms were incubated in medium supplemented with 10 μM EdU (Sigma-Aldrich, USA; catalog no. 900584) for 16 hours. The following day, worms were anesthetized and fixed as described above. EdU incorporation was detected via click chemistry using azide-fluor 545 conjugate (Sigma-Aldrich, USA; catalog no. 760757).

WISH and Fast Blue BB–stained samples were imaged using a Zeiss AxioZoom V16 light microscope (Zeiss, Germany). Fluorescent images were acquired using a Nikon A1 laser scanning confocal microscope (Nikon, Japan).

### RNA extraction and quantitative real-time PCR

For qPCR analyses, worms were collected in 1 ml of TRIzol reagent and homogenized completely. Total RNA was extracted using chloroform phase separation and precipitated with isopropanol. Reverse transcription was performed using the Evo M-MLV RT Kit (AG, China; catalog no. 11705) to synthesize cDNA from total RNA. qPCR was carried out using the SYBR Green Premix Pro Taq HS qPCR Kit (AG, China; catalog no. 11701) on a LightCycler 96 real-time PCR system (Roche, USA). *Proteasome beta type 4* (*psb4*, *Smp_056500*) was used as the endogenous reference gene for normalization. Primer sequences used for amplification are listed in table S2. Amplification efficiencies were determined for all primer pairs, and relative expression levels were subsequently calculated using the Pfaffl method for efficiency correction.

### RNA-seq and data analysis

To examine gene expression changes following *Smp_169260.1* RNAi, virgin females from single-sex infections were placed into 12-well plates with 25 worms per well and cultured in 3 ml of A169 medium supplemented with dsRNA (30 μg/ml) and 50 μM BATT. Media and dsRNA were replaced every other day until harvest at D6. As controls, worms cultured in parallel were treated with a control dsRNA. Each sample included ~50 virgins and three biological replicates were performed in each group. Worms were collected and washed with PBS two times, followed by flash freezing in liquid N_2_ for mRNA extraction and Illumina PE150 sequencing (Novogene, China). Furthermore, the RNA-seq datasets of virgins before and after pairing with male worms/treatment with BATT were generated in prior work and constitute published data downloaded from the National Center for Biotechnology Information (NCBI) Gene Expression Omnibus database (GSE184849 and GSE191062) (*7*). Reads were mapped onto the *S. mansoni* genome (WBPS19 assembly, version 10) using StringTie version 2.1.7 (*64*).

Differential gene expression analysis between experimental groups was conducted using the DESeq function within the DESeq2 framework (*65*). Genes meeting the thresholds of *P*.adj < 0.05 and Log_2_ fold change (|Log_2_FC|) ≥ 1 were designated as differentially expressed, unless otherwise specified in particular contexts. Results of differential expression analyses were visualized using the ggplot2 package (*66*).

### Astral DIA quantitative proteomics detection and analysis

Protein was extracted from ~100 virgin parasites per sample using SDT buffer (100 mM NaCl and 1% dithiothreitol) and ultrasonicated on ice for 5 min. Lysates were centrifuged (12000*g*, 15 min, 4°C) and supernatants were heated at 95°C for 10 min, cooled on ice and alkylated with iodoacetamide (1 hour, dark). Proteins were precipitated with four volumes of prechilled acetone (−20°C for ≥30 min), centrifuged (12000*g*, 15 min, 4°C), washed with cold acetone, and resuspended in dissolution buffer (6 M urea and 100 mM TEAB, pH 8.5). The concentrations were determined using the Bradford assay.

Each sample was adjusted to 100 μl with dissolution buffer and digested with trypsin at 37°C for 4 hours. Digests were acidified with formic acid (pH < 3), centrifuged (12000*g*, 5 min), and desalted using a C18 column. Peptides were washed with 0.1% formic acid/99% water and eluted with 0.1% formic acid/70% acetonitrile. Lyophilized eluates were reconstituted in 10 μl of 0.1% formic acid, centrifuged (14000*g*, 20 min, 4°C) and 200 ng was injected into a Vanquish Neo UHPLC (Thermo Fisher Scientific) with a C18 trap (5 mm by 300 μm, 5 μm) and a PepMap Neo analytical column (150 μm by 15 cm, 2 μm) at 50°C. Peptides were separated and analyzed on an Orbitrap Astral (Thermo Fisher Scientific) in data-independent acquisition (DIA) mode [mass/charge ratio (*m*/*z*) 380 to 980, 240,000 resolution, AGC 500%, 2-Th isolation, 300 windows, normalized collision energy (NCE) 25%, tandem mass spectrometry range *m*/*z* 150 to 2000, 80000 resolution, 3-ms injection].

Protein sequences from WormBase ParaSite (WBPS19) were digested in silico and a predicted spectral library was generated using DIA-NN (*67*). Raw files were aligned with automatic mass deviation correction, fixed cysteine alkylation, variable N-terminal methionine loss, and up to two missed cleavages. Peptides with Global.Q.Values < 0.01 and proteins with PG.Q.Values < 0.01 were retained. Differentially expressed proteins were defined by Student’s *t* test (*P* < 0.05, |Log_2_FC| ≥ 0.5).

### scRNA-seq and data analysis

Virgins were cultured in A169 medium with either 50 μM BATT or 0.05% DMSO (vehicle control) for 2 days. For each condition, three biological replicates (~200 worms) were processed. Single-cell suspensions were prepared as described (*8*), and only samples with >85% (fluorescence-based counting) were used. Suspensions were adjusted to 1000 cells/μl, and ~10000 cells per sample were loaded onto the 10x Genomics Chromium platform. Libraries were prepared and sequenced on an Illumina HiSeq PE150 platform (Genergy, China).

Reads were quality checked with FastQC, filtered, and processed with Cell Ranger (*68*) for genome alignment, barcode demultiplexing, PCR duplicate removal, and unique molecular identifier (UMI)–based gene barcode matrices generation. Cell-containing partitions were identified as barcodes with UMI counts >10% of the 99th percentile, separating RNA-high droplets from background RNA-low droplets. Data analysis in Seurat excluded cells with <200 or >3000 genes, <200 or >7500 total transcripts (UMIs), or >10% mitochondrial transcripts. Samples were integrated using 2000 highly variable genes and integration anchors. Normalization, principal components analysis (PCA; 50 principal components), and clustering (resolution = 1.6) followed standard Seurat workflows. Cell clusters were annotated using canonical markers (*21*, *37*). DESeq2 was applied to pseudobulk profiles, with differential expression defined as |Log_2_FC| ≥ 1 and *P*.adj < 0.05.

Approximately 60 adult females, freshly perfused from mice at 7 weeks postinfection, were processed for scRNA-seq. For analysis, cells with fewer than 200 or more than 4000 detected genes, fewer than 200 or more than 25000 transcripts, or greater than 10% mitochondrial gene content were excluded. Downstream processing involved the identification of 3000 highly variable genes, and implementation of standard workflows for data normalization, dimensionality reduction (PCA with 300 principal components), and clustering (resolution = 1).

### DAP-seq and data analysis

DAP-seq was performed as described (*69*). The DBD of Smp_169260.1 was fused to a Halo tag and expressed in vitro (wheat germ cell-free system, Promega, USA). Genomic DNA from adult female *S. mansoni* was extracted (Mollusc DNA Kit, Omega, USA) and prepared as a sequencing library (TLX DNA-seq kit, MICH, China).

Halo-tagged proteins were immobilized on Magne HaloTag beads (Promega, USA) prewashed with PBS/5% NP-40, incubated with a protein expression mixture at room temperature (RT) for 1 hour, and washed. DNA capture was performed with 100 ng of the library (1 hour, RT, rotation), followed by washing, elution (98°C, 10 min), and PCR amplification (20 cycles). Amplicons (200 to 400 bp) were gel-purified and quantified. Libraries were sequenced on an Illumina NovaSeq platform (PE150; Bluescape, China), yielding ~3 Gb per sample (~8.2× genome coverage).

The reads were quality-filtered with fastp version 0.24.0 (*70*), aligned to the *S. mansoni* reference genome (BWA-MEM v0.7.18) and peaks called with MACS version 2.2.9.1 (*71*). Peaks were associated with ChIPseeker package (*72*) into promoter (≤3 kb upstream transcription start sites), exon, intron, downstream, distal intergenic, 5′ UTR, and 3′ UTR categories. Motif enrichment and logo generation were performed with MEME-chip version 5.5.6 (*73*) and genome-wide peak distribution was visualized in IGV (*74*).

### Expression and purification of the recombinant DBD of Smp_169260.1

The DBD of Smp_169260.1 (amino acids 561 to 741) was expressed as a His-tagged recombinant protein in *E. coli* BL21 (DE3) cells using the pET-30a expression vector. Following induction, cells were harvested and lysed, and the recombinant protein was purified by nickel affinity chromatography using a HisTrap column (Cytiva, Switzerland). In brief, clarified cell lysates were loaded onto a HisTrap column pre-equilibrated with binding buffer composed of 50 mM tris-HCl, 300 mM NaCl, and 20 mM imidazole (pH 8.0). Bound proteins were eluted with an elution buffer containing 50 mM tris-HCl, 300 mM NaCl, and 500 mM imidazole (pH 8.0). Eluted fractions were analyzed by SDS–polyacrylamide gel electrophoresis, followed by Coomassie Brilliant Blue staining to assess protein purity. Western blotting using an anti-His antibody was performed to confirm the identity of the recombinant protein. The purified protein was concentrated using an Amicon Ultra centrifugal filter unit with a 10 kDa molecular weight cut-off (Millipore, Ireland), and the buffer was exchanged to PBS (pH 7.4). The final protein concentration was determined using the Bradford protein assay.

### Electrophoretic mobility shift assay

Fluorescein (FAM)–labeled and unlabeled complementary DNA oligonucleotides, each comprising a 15 bp core motif flanked by 5 bp sequences on both ends, were synthesized as single-stranded DNA by Genscript (China) and resuspended in TE buffer (10 mM tris-HCl and 0.1 mM EDTA, pH 8.0) at a concentration of 100 μM. DsDNA probes were generated by mixing equimolar amounts of complementary strands, heating to 94°C for 5 min, and gradually cooling to RT to allow annealing. Final concentrations of the resulting dsDNA probes were 50 μM for both FAM-labeled and unlabeled forms. For binding reactions, 1 pmol of FAM-labeled dsDNA was incubated with 1 μg of purified recombinant DBD protein in EMSA reaction buffer (Beyotime, China) in a total volume of 10 μl. Reactions were incubated at RT for 20 min. For competition assays, 100 pmol of unlabeled dsDNA probe was included to assess binding specificity. Samples were resolved on a 6% native polyacrylamide gel prepared in 1× TBE buffer (89 mM tris, 89 mM boric acid, and 2 mM EDTA, pH 8.3). Gels were pre-run at 200 V for 10 min, followed by electrophoresis of samples at 200 V for approximately 25 min. Fluorescence signals were visualized using a Typhoon FLA 9000 imaging system (GE Healthcare, USA).

### Dual-luciferase reporter assay

An artificial transcription factor was constructed by fusing the DBD of Smp_169260.1 with the VP64 transcriptional activation domain (comprising four tandem copies of the herpes simplex virus VP16 activation domain) and the nuclear localization signal (NLS). The promoter of *Smp_349410* was cloned upstream of the luciferase reporter in pGL4.23[luc2-minP]. Human embryonic kidney (HEK) 293T cells were cotransfected with this reporter, the expression plasmid pcDNA3.1-His-Halo-*Smp_169260.1* DBD-VP64-NLS, and pRL-TK (internal control). A His-Halo-GLI-1 DBD-VP64-NLS plasmid served as a specificity control [GLI-1 (glioma-associated homolog-1), Smp_266960].

HEK293T cells were maintained in high-glucose Dulbecco’s modified Eagle’s medium supplemented with 10% (v/v) fetal bovine serum at 37°C in a humidified atmosphere containing 5% CO_2_. Upon reaching 80 to 90% confluence, cells were detached using 0.25% trypsin-EDTA, resuspended, and seeded into 48-well plates at 500 μl per well. After 24 hours, cells were transfected with the indicated plasmids using TransIT-X2 reagent (Mirus Bio, USA) in opti-MEM, following the manufacturer’s protocol. Forty-eight hours post-transfection, media were removed, and cells were lysed with 100 μl of passive lysis buffer per well for 5 min at RT. Aliquots of 20-μl cell lysates were transferred to white 96-well plates, and firefly luciferase activity was measured following the addition of 100 μl of firefly substrate. Renilla luciferase activity was then quantified by injecting 100 μl of renilla substrate, using the Dual-Luciferase Reporter Assay System (Promega). Relative luciferase activity was obtained by calculating the ratio of firefly to Renilla luciferase activity, minimizing inherent experimental variability caused by differences in cell number, cell viability, and transfection/lysis efficiency.

### Phylogenetic analysis

Protein sequences were retrieved from NCBI and SmedGD. Alignment was conducted with MAFFT (https://ebi.ac.uk/jdispatcher/msa/mafft?stype=protein). Informatic sites were extracted using Gblock 0.91b (http://phylogeny.lirmm.fr/phylo_cgi/one_task.cgi?task_type=gblocks) for constructing phylogenetical tree in PhyML (http://www.atgc-montpellier.fr/phyml/). Substitution model was selected by SMS (*75*) in PhyML based on Bayesian Information Criterion. Branch support was validated via 1000 bootstrap replicates. Tree was visualized using FigTree version 1.4.4.

### Protein structural comparison

The predicted 3D structure of Smp_349410 was modeled using Phyre2 (https://www.sbg.bio.ic.ac.uk/phyre2/html/page.cgi?id=index). Alignment with its human homolog *Hs*CPEB1 was performed in Protein Data Bank (https://rcsb.org/alignment).

### Poly(A) tail length assay

Total RNA was extracted from approximately 50 virgins per sample using the RNeasy Micro Kit (Qiagen, Germany). Poly(A) tail length of cyclin B1 mRNA was assessed using the Poly(A) Tail-Length Assay Kit (Thermo Fisher Scientific, USA), according to the manufacturer’s protocol. In brief, G/I tailing of total RNA was performed before reverse transcription. Two sets of PCR reactions were carried out to distinguish total transcript length and poly(A) tail contribution. Fragment S was amplified using a *ccnb1*-specific forward primer (5′-TGTCTCAGTCTGTTTATTTTCCCG-3′) and a *ccnb1*-specific reverse primer (5′-ACACTAAAACCGAAGACGATCA-3′) located 30-nt upstream of the poly(A) start site. Fragment A was amplified using the same gene-specific forward primer and a 35-nt universal reverse primer (provided in the kit) that binds to the G/I-tailed cDNA terminus. PCR products were separated on a 2.5% agarose gel with GelRed and visualized by gel electrophoresis. Poly(A) tail length was estimated by subtracting the size of Fragment S from that of Fragment A and was quantified on the basis of band intensity and migration distance using ImageJ software.

### Statistical analysis

All statistical analyses were performed using GraphPad Prism software (version 9.3.1 for Windows; GraphPad Software, San Diego, CA, USA). A significance level of 0.05 was used throughout the study. The normality of each dataset was first assessed using the Shapiro-Wilk test. For normally distributed data, either paired-samples or independent-samples *t* tests were applied, depending on the experimental design. For non-normally distributed data, the Wilcoxon signed-rank test (for paired samples) or the Mann-Whitney *U* test (for independent samples) was used. When multiple comparisons were conducted, *P* values were adjusted using the Bonferroni correction. Statistical significance is denoted as follows: ns (not significant), *P* < 0.05 (*), *P* < 0.01 (**), *P* < 0.001 (***), and *P* < 0.0001 (****).
