# Supplementary material for: A male-pheromone-elevated transcription factor ZNF362.1 in female schistosomes determines sexual maturation
Source: Sci Adv. 2026 Mar 6;12(10):eaec6907. doi: 10.1126/sciadv.aec6907 (PMC12965309; doi:10.1126/sciadv.aec6907)
Supplement: Supplementary file 1 — Figs. S1 to S21 Legends for tables S1 to S16 [file sciadv.aec6907_sm.pdf]

Science Advances
AAAS

Supplementary Materials for

A male-pheromone-elevated transcription factor ZNF362.1 in female schistosomes determines sexual maturation

Mengjie Gu *et al.*

Corresponding author: Jipeng Wang, jipengwang@fudan.edu.cn

Sci. Adv. **12**, eaec6907 (2026)
DOI: 10.1126/sciadv.aec6907

The PDF file includes:

Figs. S1 to S21
Legends for tables S1 to S16

Other Supplementary Material for this manuscript includes the following:

Tables S1 to S16

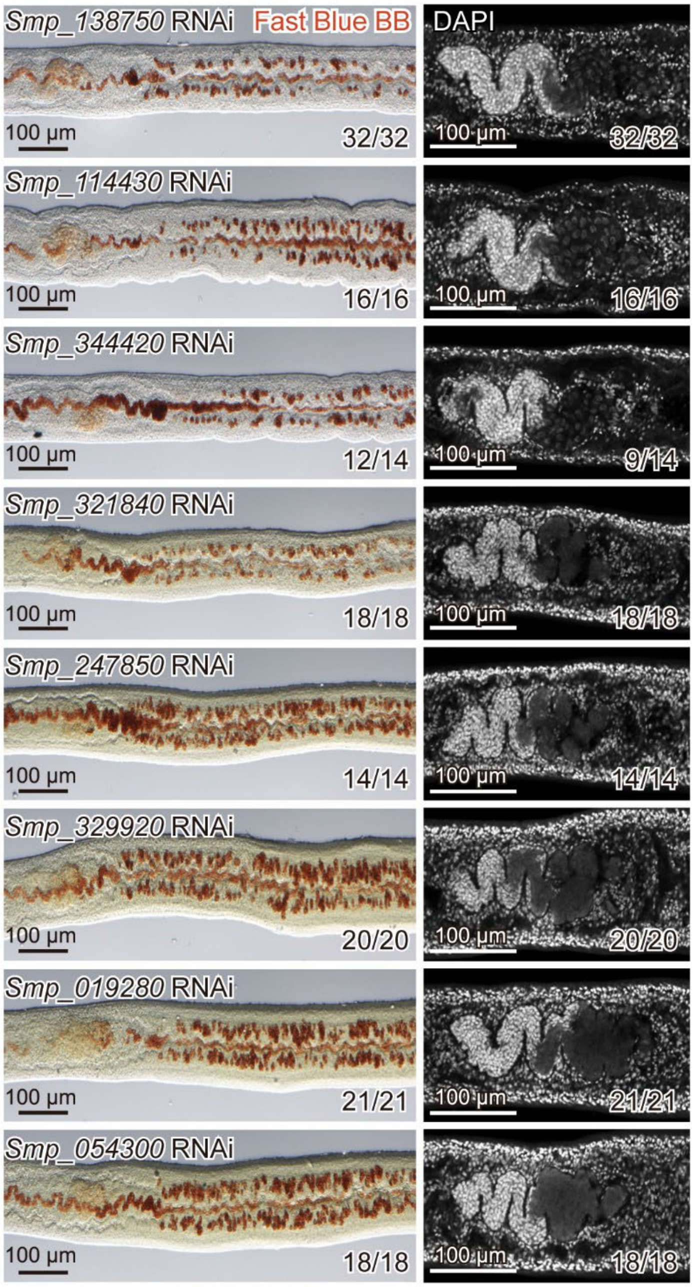

fig. S1. Functional validation of eight genes upregulated in response to male pheromone stimulation during the sexual development of virgin schistosomes.

Virgin females were treated with gene-specific dsRNA for 10 days in A169 medium supplemented with BATT, followed by Fast Blue BB staining (left) to visualize vitellaria and DAPI staining (right) to label the nuclei of ovaries. *N* = 3. Scale bars = 100 µm.

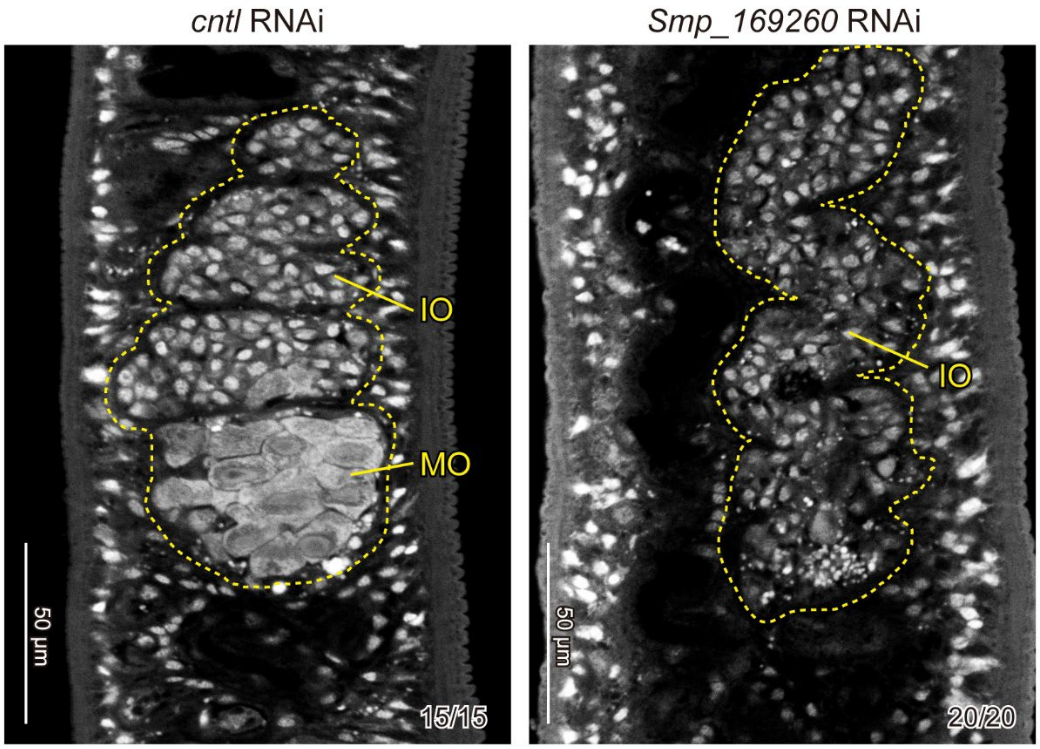

fig. S2. Confocal microscopy of ovaries from female schistosomes following *Smp_169260* RNAi.

After 10 days of dsRNA treatment, female worms were stained with hydrochloric carmine to visualize ovarian structures. IO, immature oocytes; MO, mature oocytes. *N* = 3. Scale bars = 50 µm.

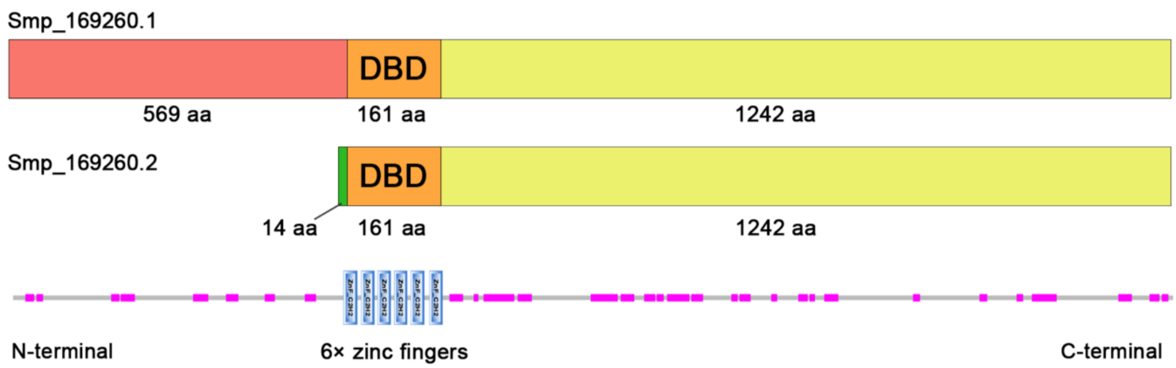

fig. S3. Structural comparison of the two protein isoforms encoded by *Smp_169260*.
Schematic diagram illustrating the sequence differences between the protein products of *Smp_169260.1* and *Smp_169260.2*.

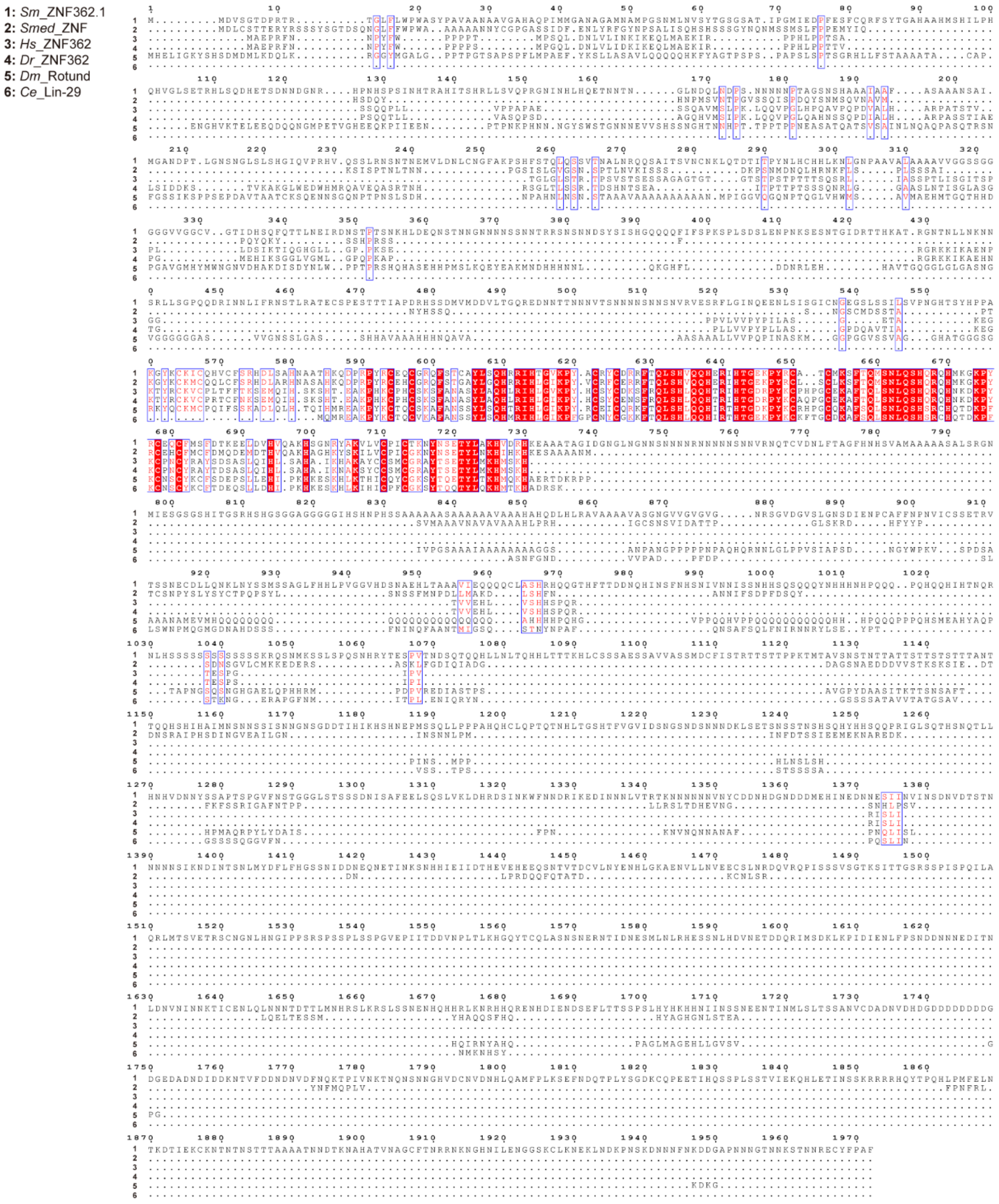

fig. S4. Protein sequence alignment of Smp_169260 with homologs from model species.

Conservation was assessed based on physicochemical properties. Columns with a similarity score > 0.7 are defined as highly conserved and are highlighted in red with a blue frame. Strictly identical residues are shown in white on a red background. Homologs of Smp_169260 includes zinc finger protein (SmedGD: SMED30013184-orf-1) from *Schmidtea mediterranea* (BLAST E-value = $9e^{-107}$), zinc finger protein 362 (ZNF362, GenBank: XP_005270564.1) from *Homo*

sapiens (BLAST E-value = $8e^{-49}$), ZNF362 (GenBank: NP_001083017.1) from *Danio rerio* (BLAST E-value = $3e^{-49}$), Rotund (GenBank: NP_001138021.1) from *Drosophila melanogaster* (BLAST E-value = $6e^{-47}$), and Lin-29 (GenBank: NP_001407504.1) from *Caenorhabditis elegans* (BLAST E-value = $6e^{-44}$).

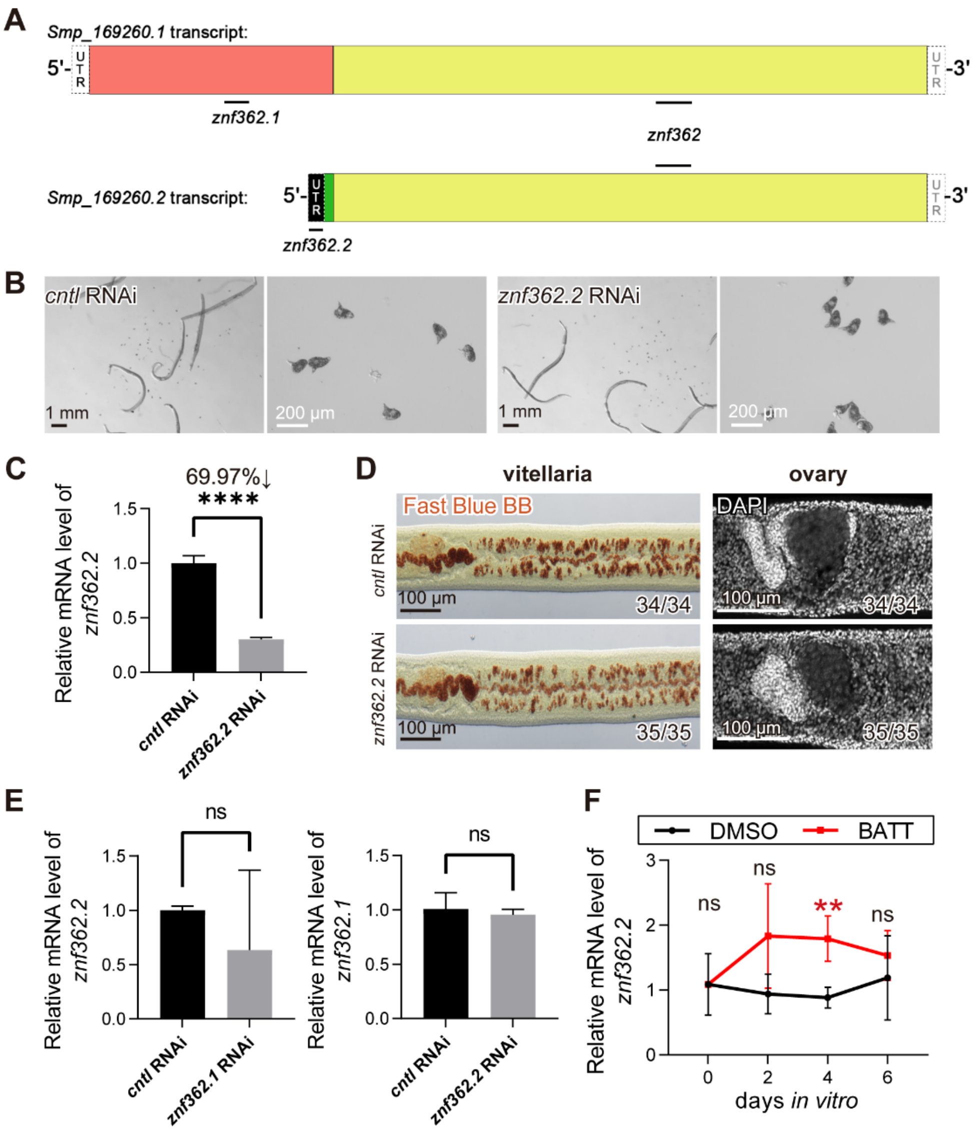

fig. S5. The short transcript *znf362.2* is not required for sexual development in virgin female schistosomes.

(**A**) Schematic diagram illustrating the sequence differences between the transcripts of *znf362.1* and *znf362.2*. Three dsRNA target sites for common or transcript-specific knockdown are indicated. (**B**) Light microscopy images showing the morphology and oviposition status of virgin females following 10 days of *in vitro* treatment with control or *Smp_169260.2* dsRNA. Left panels: whole-worm morphology (scale bars = 1 mm); right panels: egg morphology (scale bars = 200 μm). $N = 3$. (**C**) qPCR quantification of *znf362.2* transcript levels at D10 in control and RNAi-treated groups (****$P < 0.0001$). Data are presented as mean ± SD. $N = 3$. (**D**) Fast Blue BB and DAPI staining of vitellaria and ovaries in control and *Smp_169260.2*-RNAi virgin females after 10 days of treatment. Fast Blue BB staining of vitellaria (left); DAPI staining of ovaries (right). IO, immature oocytes; MO, mature oocytes. $N = 3$. Scale bars = 100 μm. (**E**) qPCR quantification of *znf362.2* transcript levels at D10 in control and *znf362.1* RNAi groups (left) and qPCR quantification of *znf362.1* transcript levels at D10 in control and *znf362.2* RNAi groups (right). (**F**) qPCR quantification of *znf362.2* transcript levels from D0 to D6 following

BATT induction. The black line indicates the mRNA abundance in the DMSO control group, while the red line represents the BATT-induced group. $^{**}P < 0.01$. Data are presented as mean ± SD. $N = 3$. Unpaired Student's t test was applied for (C) and (E).

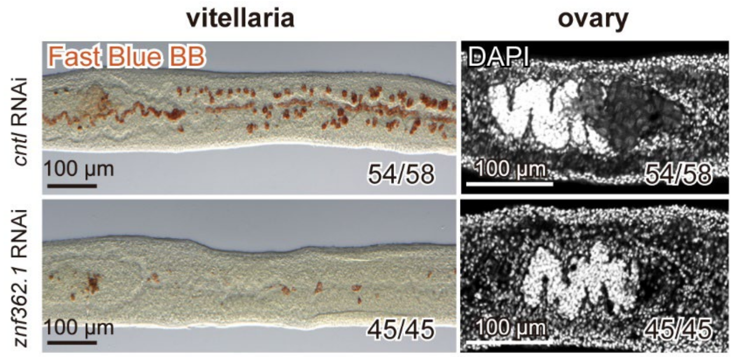

fig. S6. Fast Blue BB and DAPI staining of vitellaria and ovaries from control and *znf362.1*-RNAi virgin females following BATT induction.

Virgin females were subjected to for 6 days RNAi during 10 days BATT induction. Fast Blue BB staining of vitellaria (left); DAPI staining of ovaries (right). *N* = 3. Scale bars = 100 μm.

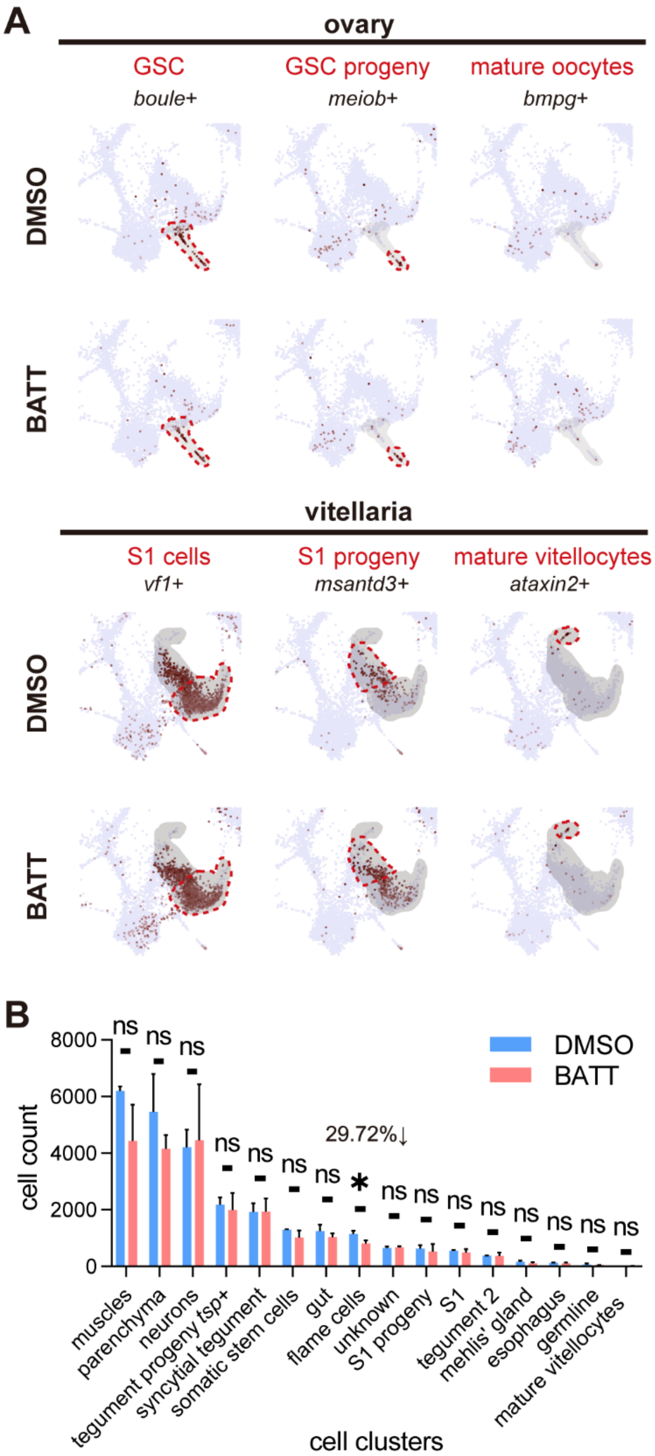

fig. S7. Ovary and vitellaria populations in the scRNA-seq atlas of virgin female schistosomes.

(**A**) UMAP plots showing cell clusters expressing well-defined ovary- and vitellaria-associated marker genes in virgin females after 2 days of DMSO or BATT treatment. Higher expression levels are indicated by darker brown color. (**B**) Quantitative comparison of each cell population in virgin females treated with BATT for 2 days versus DMSO controls. Statistical significance

was assessed using a *t*-test (*$P < 0.05$). Data are presented as mean ± SD. $N = 3$. Unpaired Student's *t* test and Mann–Whitney *U* test are applied for (B) according to the normality of each dataset.

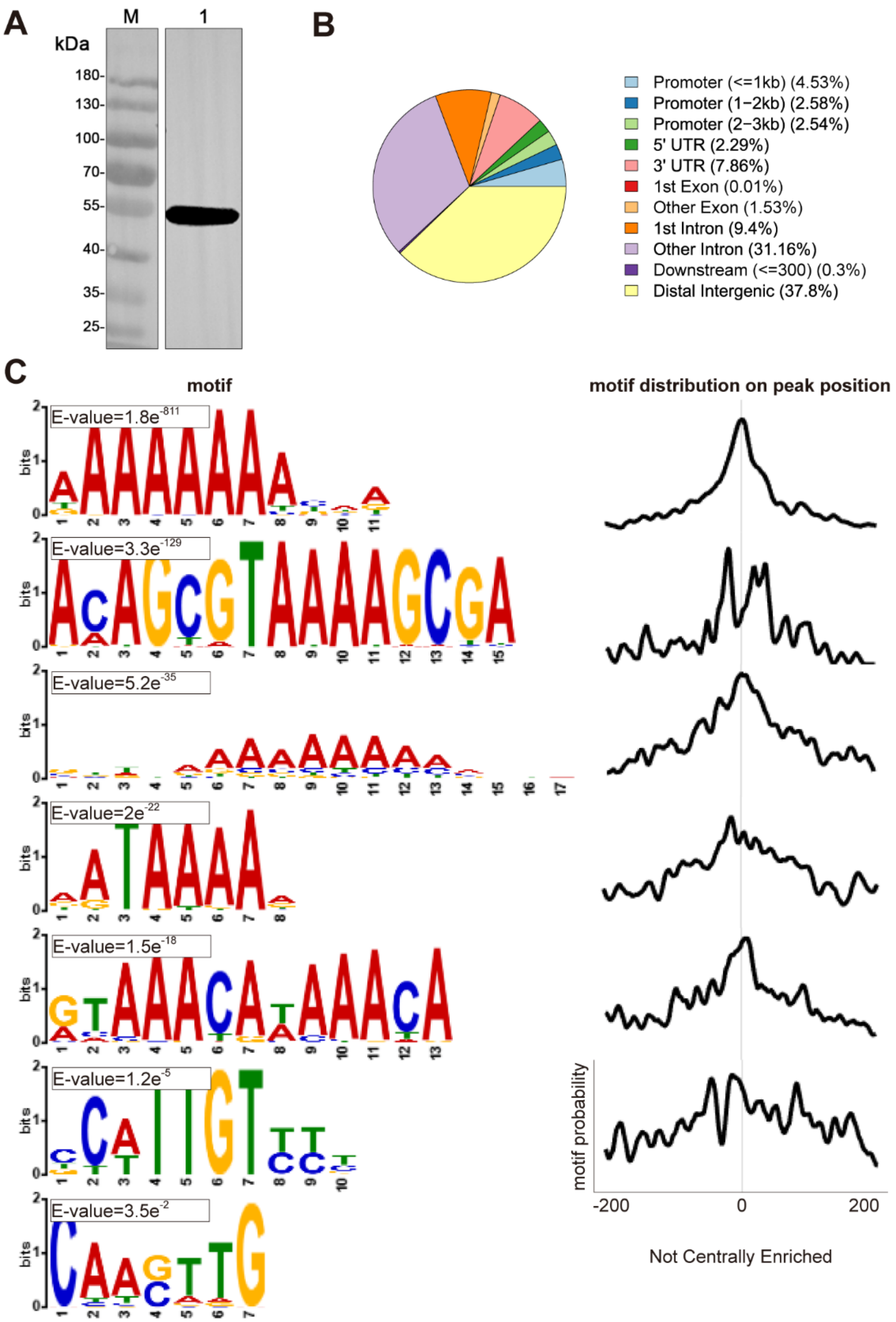

fig. S8. Genomic annotation and motif analysis of the ZNF362.1 DNA-binding domain based on DAP-seq data.

(**A**) Western blot validation of the cell-free expression of the ZNF362.1 DNA-binding domain. (**B**) Genomic distribution of peaks identified by DAP-seq for the ZNF362.1 DNA-binding

domain. (**C**) Identification of seven binding motifs for the ZNF362.1 DNA-binding domain from promoter-associated peak regions and their positional distribution within peaks.

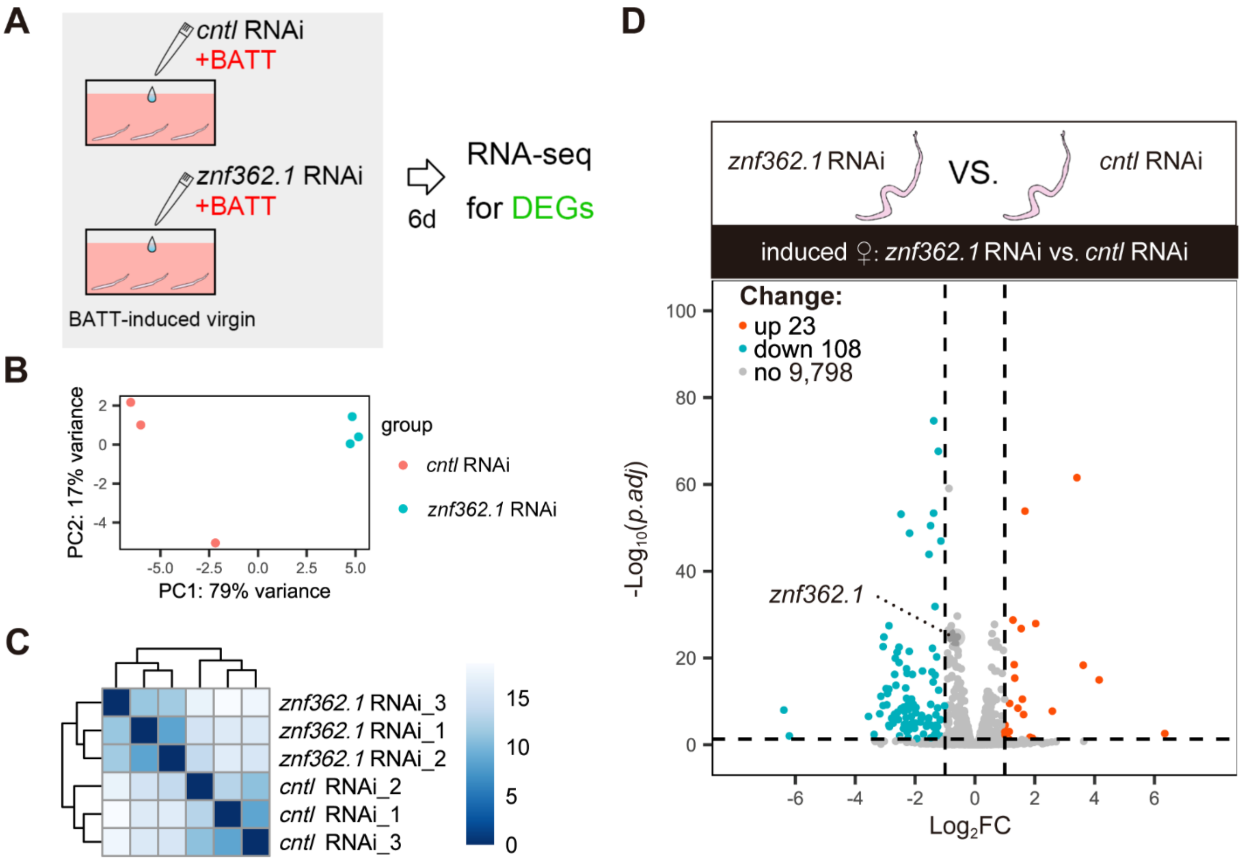

fig. S9. Transcriptomic profiles of control and *znf362.1*-RNAi virgin females following 6 days of BATT induction.

(**A**) Schematic diagram of the RNA-seq experimental design. Virgin females were treated with RNAi in A169 medium supplemented with BATT for 6 days. (**B**) Principal component analysis (PCA) of transcriptomes from control and *znf362.1*-RNAi groups. $N = 3$. (**C**) Inter-sample distance matrix showing transcriptomic similarity among samples from control and *znf362.1*-RNAi groups. (**D**) Volcano plot illustrating differential gene expression between control and *znf362.1*-RNAi females. Significantly differentially expressed genes were defined as those whose $|\text{Log}_2\text{FC}| \geq 1$ and $p.adj < 0.05$.

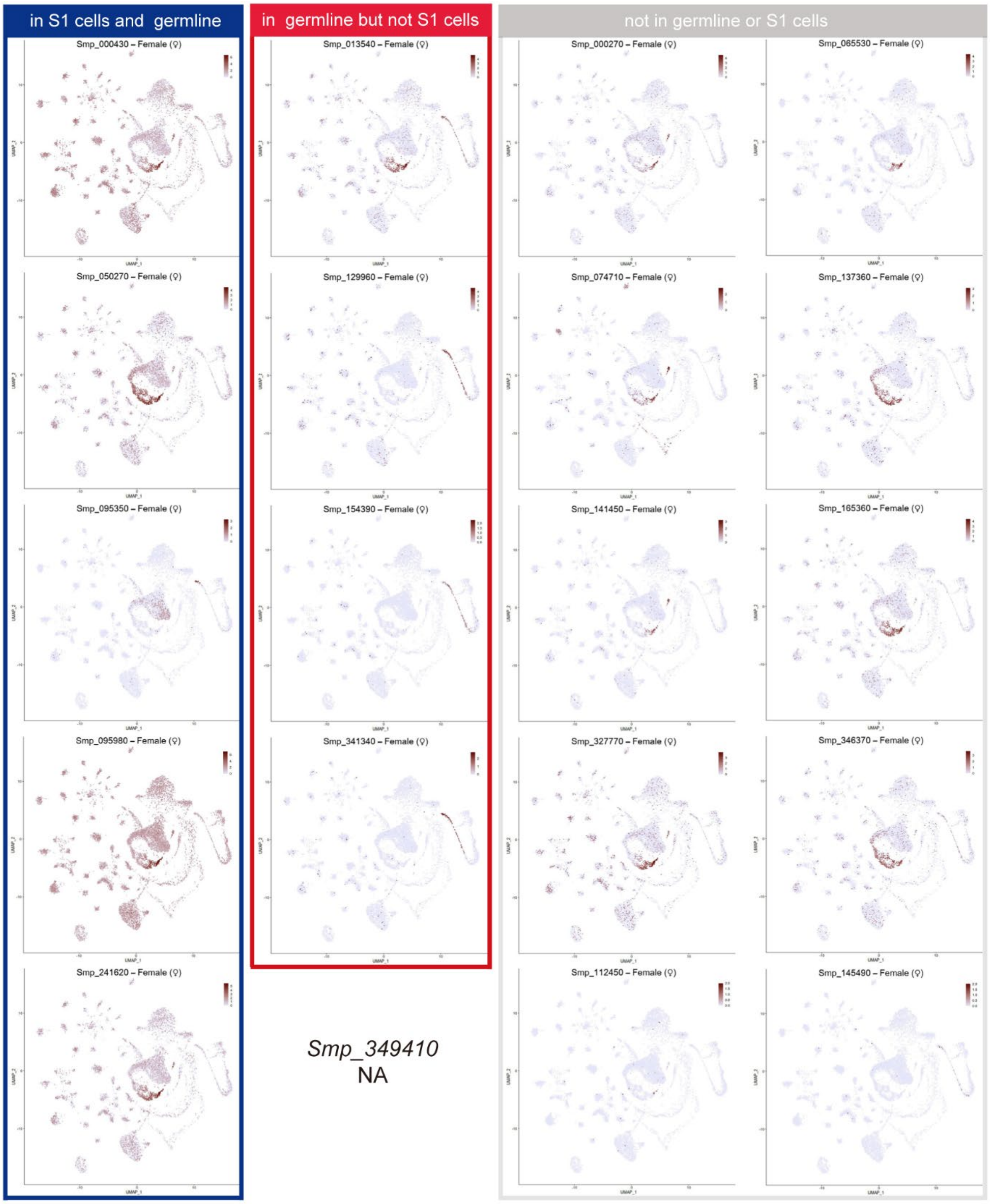

fig. S10. UMAP plots of 20 potential downstream targets of ZNF362.1.

UMAP plots of gene expression for 19 genes were obtained from the SchistoCyte Atlas (https://www.collinslab.org/schistocyte/). The UMAP plot for *Smp_349410* is not available in the SchistoCyte Atlas.

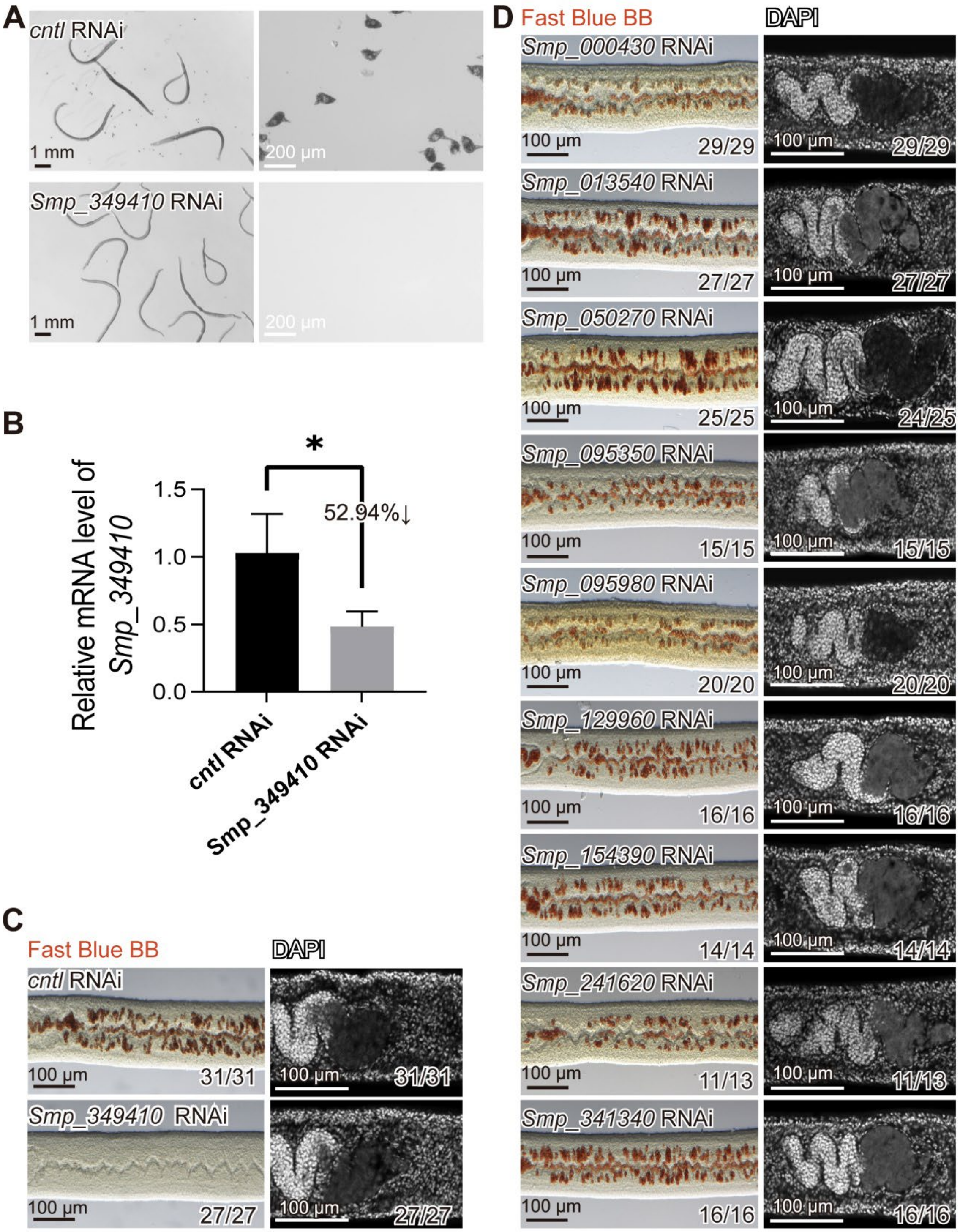

fig. S11. Functional analysis of potential ZNF362.1 downstream targets in female sexual development.

(**A**) Light microscopy images showing the morphology and oviposition status of virgin females following 10 days of *in vitro* treatment with control or *Smp_349410* dsRNA (BATT inducing from D0). Left panels: whole-worm morphology (scale bars = 1 mm); right panels: egg morphology (scale bars = 200 μm). *N* = 3. (**B**) Quantitative PCR validation of *Smp_349410*

knockdown in virgin females after 10 days of RNAi treatment (BATT inducing from D0). Data are presented as mean ± SD. *P = 0.04. N = 3. (**C**) Fast Blue BB and DAPI staining of vitellaria and ovaries in control and *Smp_349410*-RNAi virgin females after 10 days of treatment (BATT inducing from D0). Fast Blue BB staining of vitellaria (left); DAPI staining of ovaries (right). IO, immature oocytes; MO, mature oocytes. N = 3. Scale bars = 100 µm. (**D**) Functional validation of nine genes upregulated by ZNF362.1. Virgin females were treated with gene-specific dsRNA for 21 days in A169 medium (BATT inducing from D7), followed by Fast Blue BB staining (left) to visualize vitellaria and DAPI staining (right) to label the nuclei of ovaries. N = 3. Scale bars = 100 µm. Unpaired Student's t test was applied for (B).

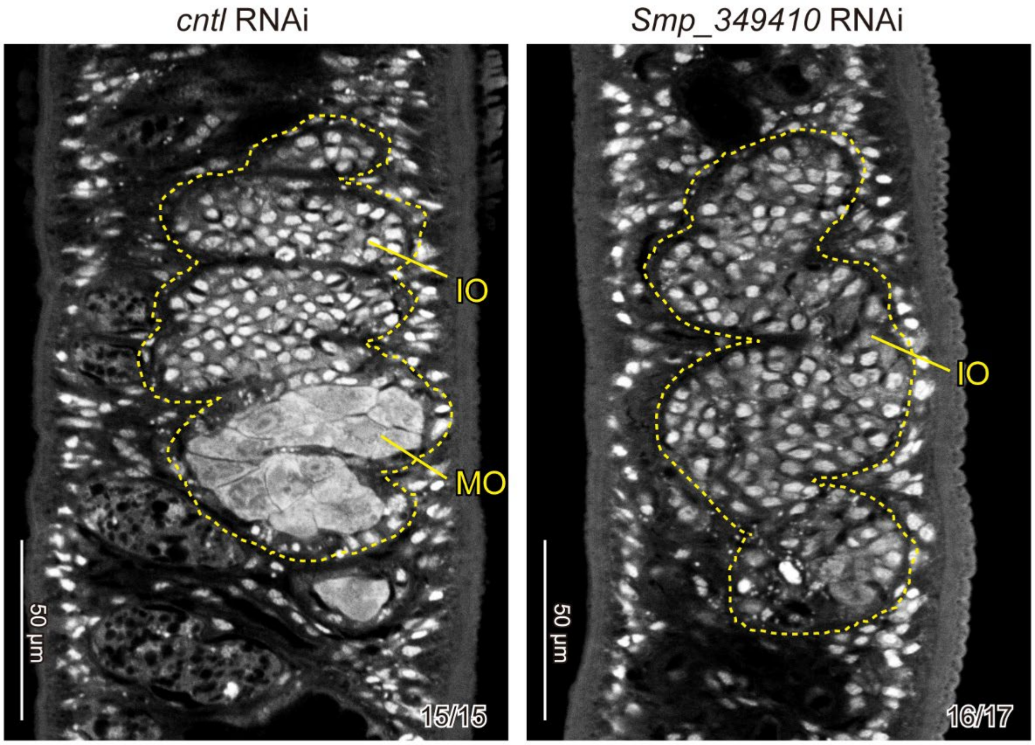

fig. S12. Confocal microscopy of ovaries from female schistosomes following *Smp_349410* RNAi.

After 21 days of dsRNA treatment (BATT inducing from D7), female worms were stained with hydrochloric carmine to visualize ovarian structures. IO, immature oocytes; MO, mature oocytes. *N* = 3. Scale bars = 50 µm.

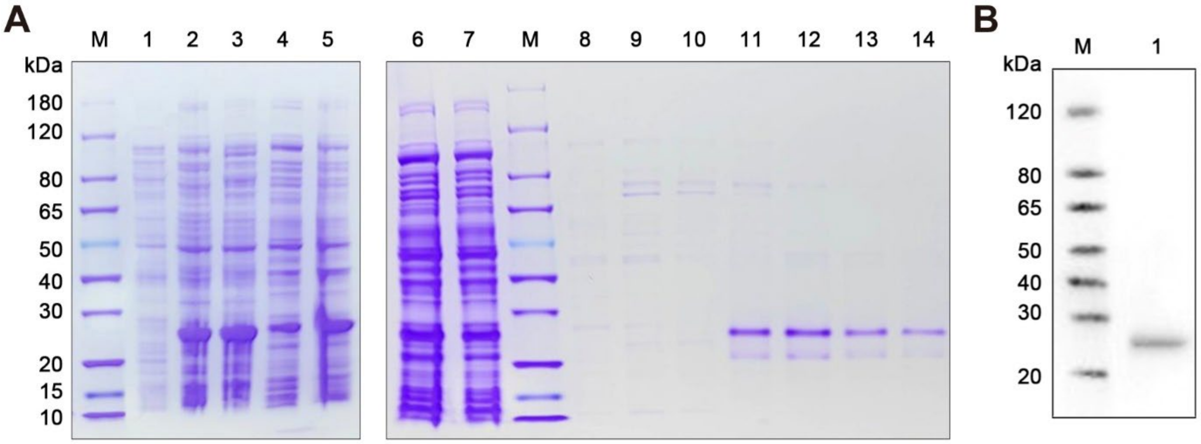

fig. S13. Expression and purification of the recombinant ZNF362.1 DNA binding domain.
(**A**) SDS-PAGE analysis of prokaryotic expression and purification of ZNF362.1 DBD. M: Protein molecular weight marker; Lane 1: Uninduced whole-cell lysate (no IPTG); Lane 2: Whole-cell lysate induced at 15°C for 16 h; Lane 3: Whole-cell lysate induced at 37°C for 16 h; Lane 4: Supernatant of lysate induced at 15°C; Lane 5: Pellet of lysate induced at 15°C; Lane 6: Pre-purification supernatant; Lane 7: Flow-through after incubation with Ni-IDA resin; Lane 8: Elution with 50 mM imidazole; Lanes 9–10: Elution with 100 mM imidazole; Lanes 11–14: Elution with 500 mM imidazole. (**B**) Western blot validation of the purified ZNF362.1 DBD using an anti-His-tag antibody.

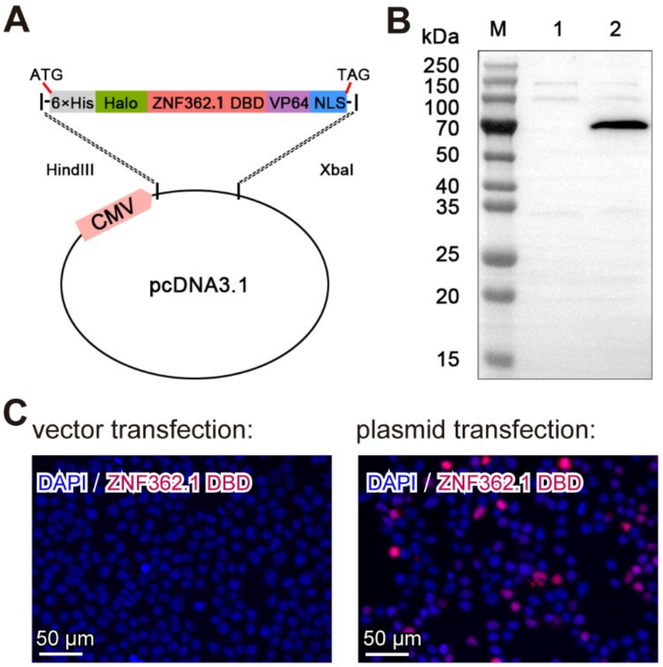

fig. S14. Recombinant expression of the ZNF362.1 DNA binding domain for the dual-luciferase assay.

(**A**) Schematic representation of the engineered transcription factor His-Halo-ZNF362.1 DBD-VP64-NLS, constructed using the ZNF362.1 DBD. (**B**) Western blot analysis confirming the expression of the engineered transcription factor in 293T cells. M: Protein molecular weight marker; Lane 1: Total protein from non-transfected cells; Lane 2: Total protein from cells transfected with the pCDNA3.1-His-Halo-ZNF362.1 DBD-VP64-NLS plasmid. (**C**) Subcellular localization of the engineered His-Halo-ZNF362.1 DBD-VP64-NLS protein in 293T cells. The ZNF362.1 DBD signal is shown in magenta by the Halo Tag fluorescent ligand; nuclei are counterstained with DAPI (blue). $N = 3$. Scale bars = 50 μm.

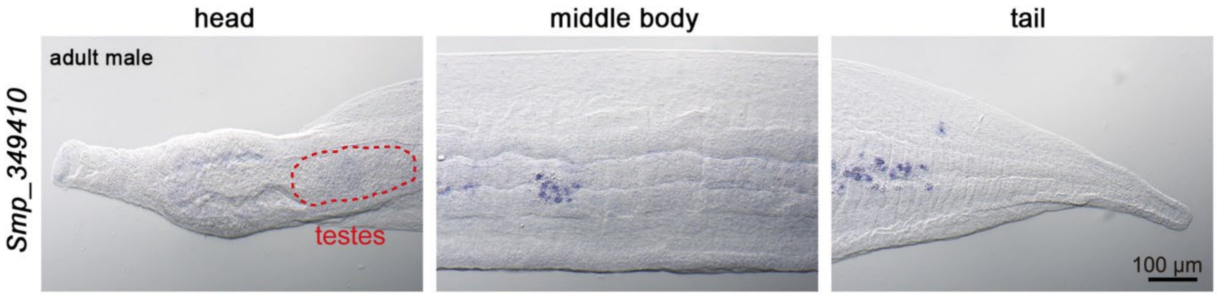

fig. S15. Whole-mount *in situ* hybridization of *Smp_349410* in adult males.

Purple staining indicates *Smp_349410* mRNA localization. Representative images of n > 12 parasites from 3 biological replicates. Scale bars = 100 µm.

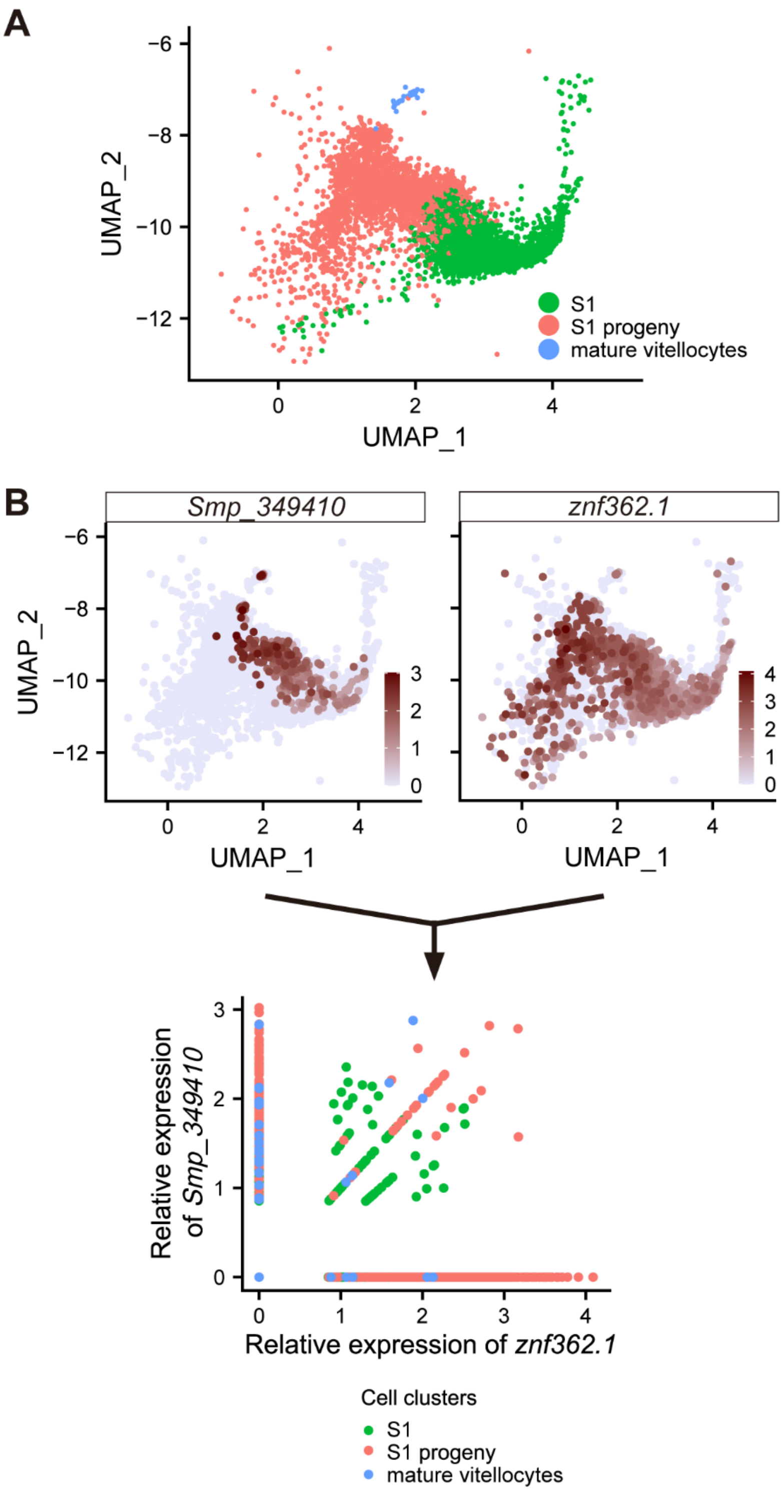

fig. S16. Co-expression of *Smp_349410* and *znf362.1* in vitellocytes.

(**A**) UMAP plot of vitellocytes. (**B**) The top panel depicts the expression distribution of *Smp_349410* or *znf362.1* across all vitellocytes; the bottom panel shows the relative expression of *Smp_349410* and *znf362.1* in each vitellocyte.

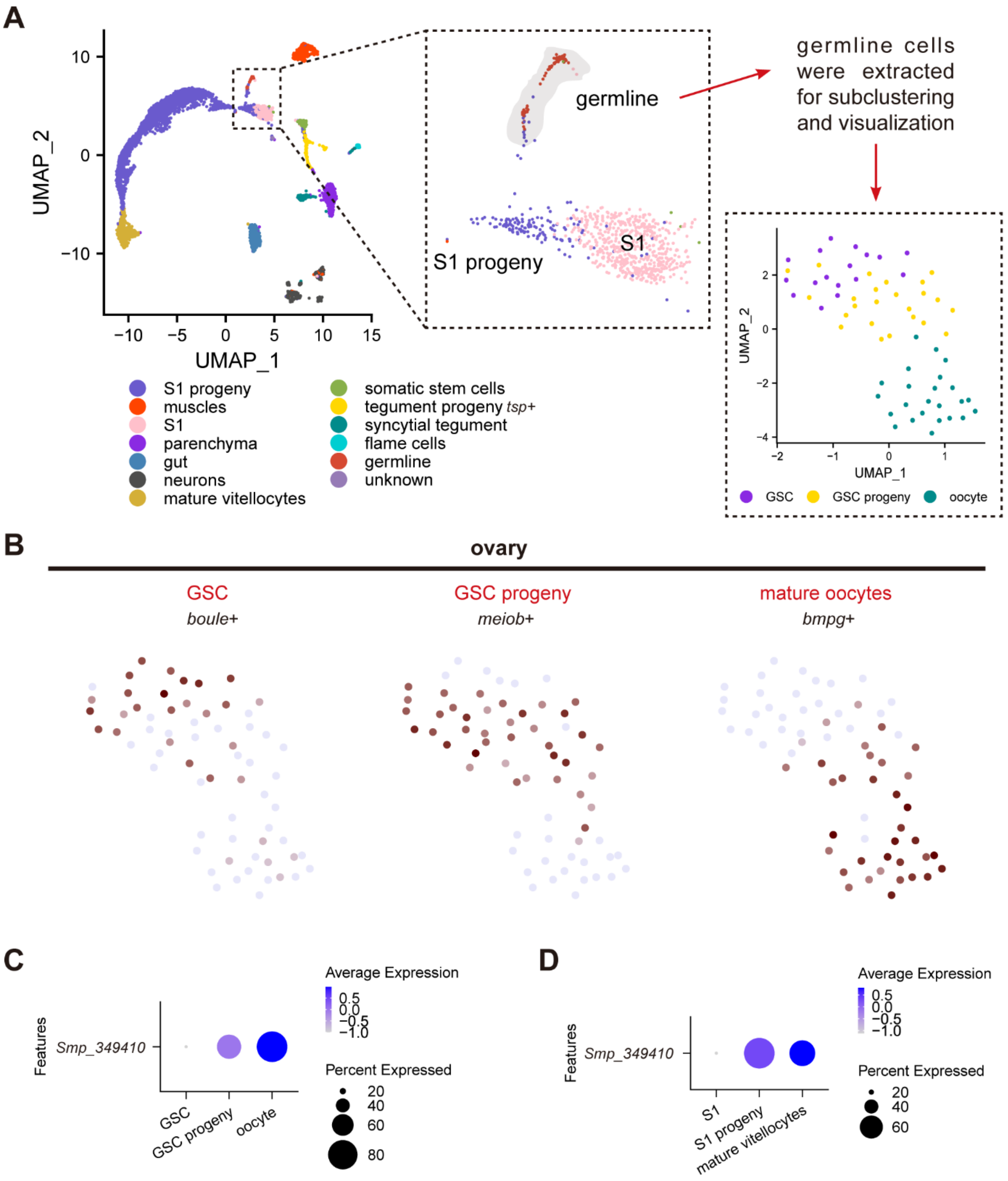

fig. S17. Single-cell resolution of *Smp_349410* expression in the scRNA-seq atlas of adult female schistosomes.

(**A**) UMAP plot of scRNA-seq data from adult females, clustered based on established cell-type marker genes. Germline cells were extracted and subclustered for more precise classification. (**B**) UMAP plots showing germline clusters expressing well-defined ovary-associated marker genes in adult females. Higher expression levels are indicated by darker brown color. (**C**) Relative

expression of *Smp_349410* across different germline cell stages in adult females. (**D**) Relative expression of *Smp_349410* across different vitellaria cell stages in adult females.

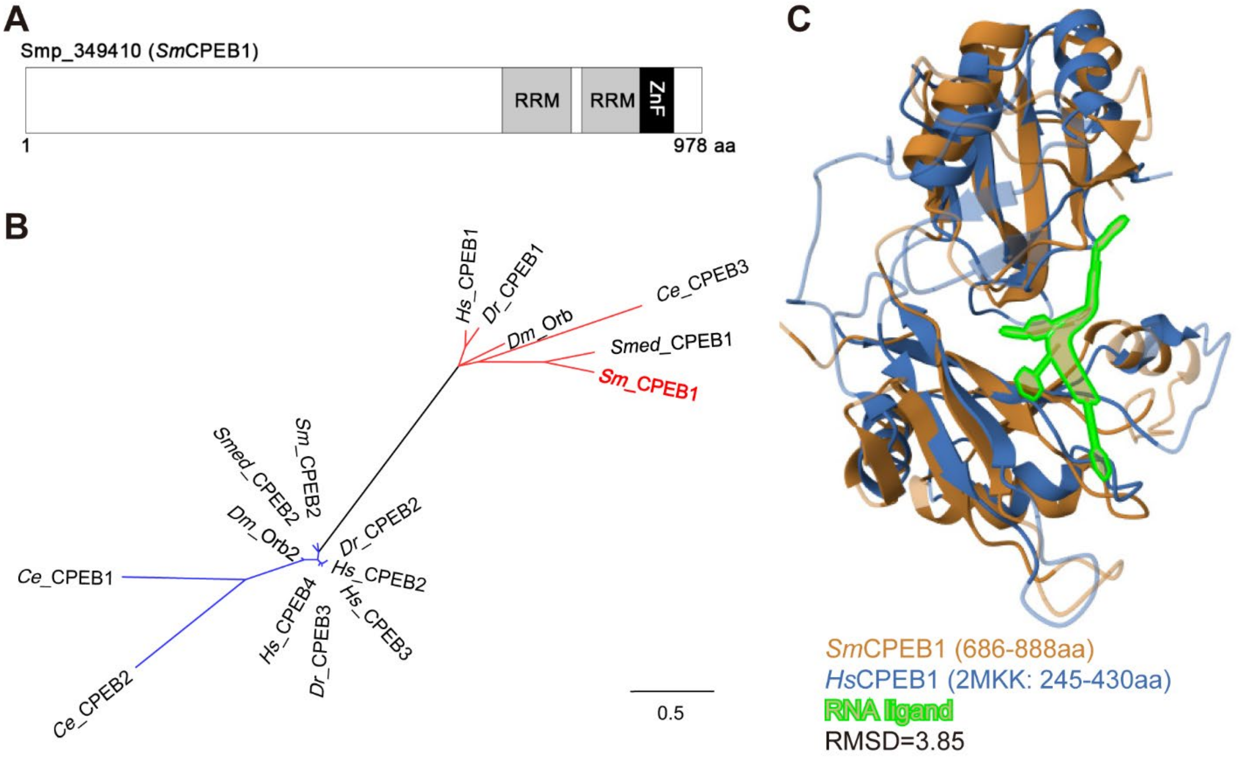

fig. S18. Protein domain architecture of *Sm*CPEB1 and its phylogenetic analysis with homologs.

(**A**) Schematic representation of the linear domain architecture of *S. mansoni* CPEB1 (Smp_349410). It contains two RNA recognition motifs (RRMs) and one zinc finger (ZnF) domain in their C-terminal regions. (**B**) Phylogenetic tree of *S. mansoni* CPEB1 and its homologs in representative model organisms based on the maximum likelihood method. *Sm*: *Schistosoma mansoni*; *Smed*: *Schmidtea mediterranea*; *Ce*: *Caenorhabditis elegans*; *Dm*: *Drosophila melanogaster*; *Dr*: *Danio rerio*; *Hs*: *Homo sapiens*. (**C**) Structural comparison of the predicted 3D structure of *Sm*CPEB1 with its human homolog *Hs*CPEB1. RNA ligand was highlighted with green border.

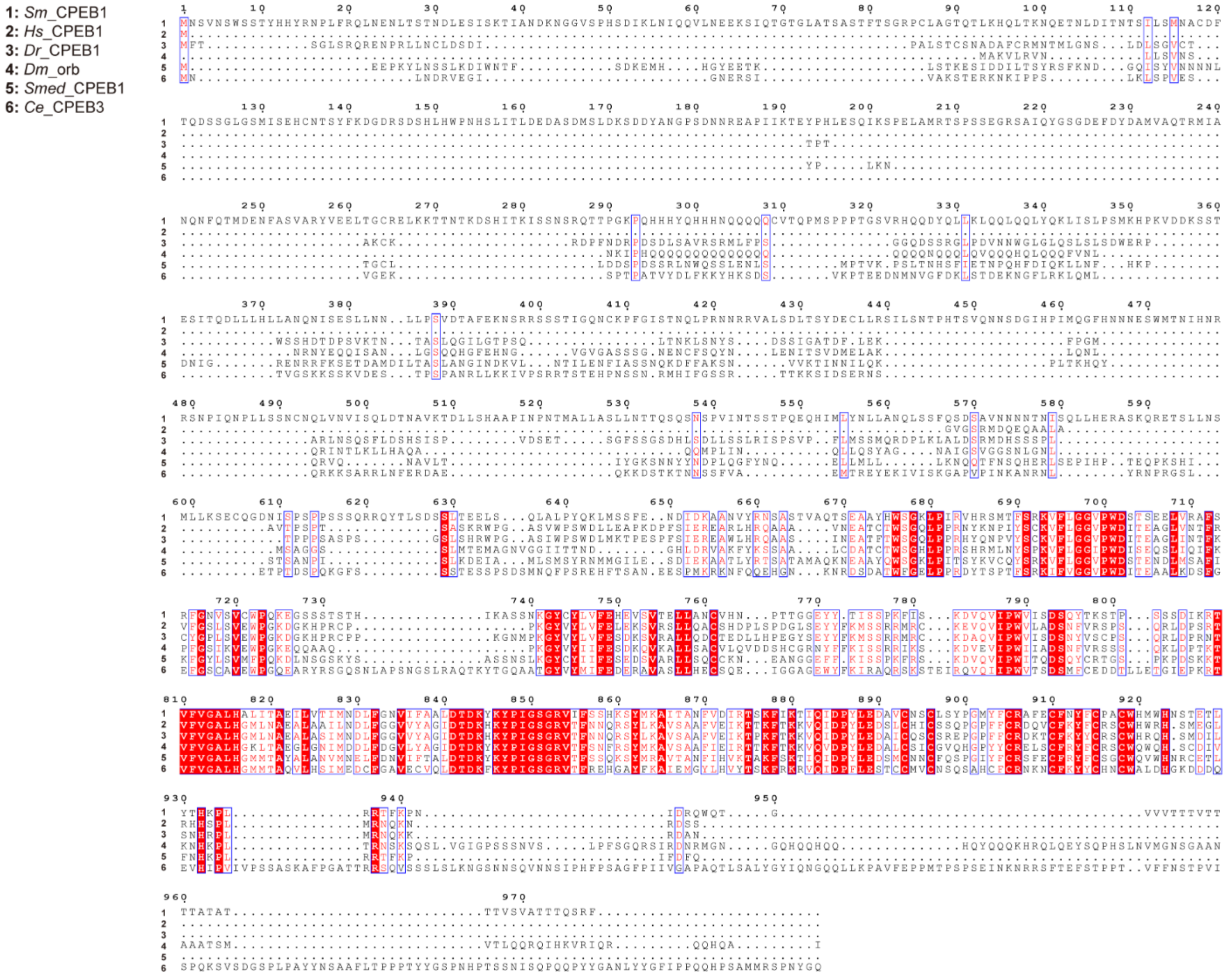

fig. S19. Protein sequence alignment of Smp_349410 with homologs from model species. Conservation was assessed based on physicochemical properties. Columns with a similarity score > 0.7 are defined as highly conserved and are highlighted in red with a blue frame. Strictly identical residues are shown in white on a red background. Homologs of Smp_349410 include zinc finger protein (GeneBank: ARM37828.1) from *Sc. mediterranea* (BLAST E-value = 1e^{-145}), cytoplasmic polyadenylation element-binding protein 1 (CPEB1, GeneBank: NP_001275749.1) from *H. sapiens* (BLAST E-value = 2e^{-91}), OO18 RNA-binding protein (Orb, GeneBank: NP_732850.1) from *Dr. melanogaster* (BLAST E-value = 1e^{-89}), CPEB1 (GeneBank: XP_068078259.1) from *D. rerio* (BLAST E-value = 8e^{-88}), and CPEB3 (GeneBank: NP_491680.2) from *C. elegans* (BLAST E-value = 4e^{-64}).

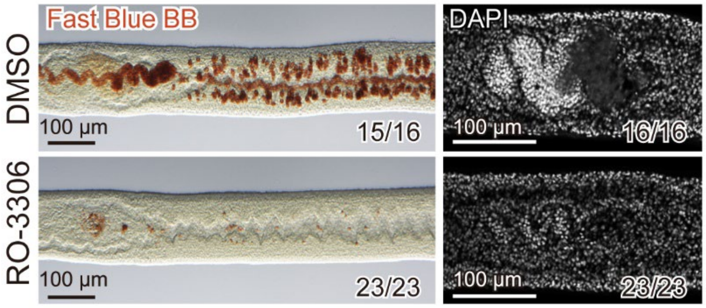

fig. S20. Fast Blue BB and DAPI staining of vitellaria and ovaries in control and RO-3306-treated virgin females following BATT induction.

Virgin females were treated with the CDK1 inhibitor RO-3306 during the 10-day BATT induction. Fast Blue BB staining of vitellaria (left); DAPI staining of ovaries (right). *N* = 3. Scale bars = 100 µm.

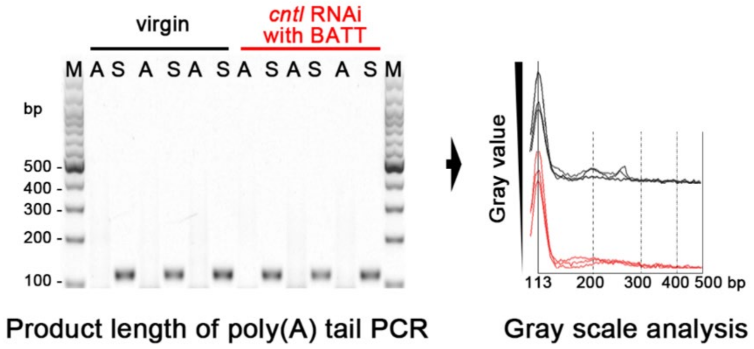

fig. S21. Poly(A) tail length analysis of cyclin B1 mRNA in somatic tissue.

Gray scale analysis of poly(A) tail PCR products from the somatic part in females from the virgin, BATT-induced control RNAi groups. A, poly(A) tail PCR product (A); S, gene-specific PCR product (S); M, 100 bp DNA Ladder. The relative poly(A) tail length was estimated from the peak summit, as indicated by the arrows. $N = 3$.

table S1. (separate file)
Primers for RNAi and *in situ* hybridization.

table S2. (separate file)
Primers for qPCR.

table S3. (separate file)
Gene lists derived from RNA-seq analysis for the identification of BATT-responsive genes.

table S4. (separate file)
RNA-seq data from virgin females paired with *gli1* RNAi males for 3d.

table S5. (separate file)
The mRNA and protein abundance of *znf362.1* in virgin females after BATT induction.

table S6. (separate file)
The mRNA and protein abundance of *znf362.1* in virgin females after *znf362.1* RNAi under BATT induction.

table S7. (separate file)
The mRNA abundance of *znf362.2* in virgin females after BATT induction.

table S8. (separate file)
List of genes significantly enriched in single-cell clusters.

table S9. (separate file)
The *znf362.1* expression across distinct cell clusters between control and BATT-treated groups at day 2.

table S10. (separate file)
Genomic annotation of ZNF362.1 binding peaks identified by DAP-seq.

table S11. (separate file)
RNA-seq data from virgin female schistosomes following *znf362.1* RNAi treatment.

table S12. (separate file)
RNA-seq data from virgin female schistosomes following 8d BATT treatment.

table S13. (separate file)
Gene lists from RNA-seq and DAP-seq analyses for identification of downstream targets of ZNF362.1.

table S14. (separate file)
The mRNA abundance of *Smp_349410* in virgin females after BATT induction.

table S15. (separate file)

The mRNA abundance of *Smp_349410* in virgin females after *znf362.1* RNAi under BATT induction.

table S16. (separate file)

Accession IDs of protein sequences used in the phylogenetic tree.
